# *Aspergillus ochraceus*: Metabolites, Bioactivities, Biosynthesis, and Biotechnological Potential

**DOI:** 10.3390/molecules27196759

**Published:** 2022-10-10

**Authors:** Rawan H. Hareeri, Mohammed M. Aldurdunji, Hossam M. Abdallah, Ali A. Alqarni, Shaimaa G. A. Mohamed, Gamal A. Mohamed, Sabrin R. M. Ibrahim

**Affiliations:** 1 Department of Pharmacology and Toxicology, Faculty of Pharmacy, King Abdulaziz University, Jeddah 21589, Saudi Arabia; 2 Department of Clinical Pharmacy, College of Pharmacy, Umm Al-Qura University, P.O. Box 13578, Makkah 21955, Saudi Arabia; 3 Department of Natural Products and Alternative Medicine, Faculty of Pharmacy, King Abdulaziz University, Jeddah 21589, Saudi Arabia; 4 Department of Pharmacognosy, Faculty of Pharmacy, Cairo University, Cairo 11562, Egypt; 5 Pharmaceutical Care Department, Ministry of National Guard—Health Affairs, Jeddah 22384, Saudi Arabia; 6 Faculty of Dentistry, British University, Suez Desert Road, Cairo 11837, Egypt; 7 Department of Chemistry, Preparatory Year Program, Batterjee Medical College, Jeddah 21442, Saudi Arabia; 8 Department of Pharmacognosy, Faculty of Pharmacy, Assiut University, Assiut 71526, Egypt

**Keywords:** *Aspergillus ochraceus*, Aspergillaceae, fungi, metabolites, bioactivities, biosynthesis, enzymes

## Abstract

Fungus continues to attract great attention as a promising pool of biometabolites. *Aspergillus ochraceus* Wilh (Aspergillaceae) has established its capacity to biosynthesize a myriad of metabolites belonging to different chemical classes, such as isocoumarins, pyrazines, sterols, indole alkaloids, diketopiperazines, polyketides, peptides, quinones, polyketides, and sesquiterpenoids, revealing various bioactivities that are antimicrobial, cytotoxic, antiviral, anti-inflammatory, insecticidal, and neuroprotective. Additionally, *A. ochraceus* produces a variety of enzymes that could have variable industrial and biotechnological applications. From 1965 until June 2022, 165 metabolites were reported from *A. ochraceus* isolated from different sources. In this review, the formerly separated metabolites from *A. ochraceus*, including their bioactivities and biosynthesis, in addition, the industrial and biotechnological potential of *A. ochraceus* are highlighted.

## 1. Introduction

Recently, a dramatic shift has increased towards the utilization of eco-friendly and sustainable sources for discovering therapeutic agents against various health concerns to promote human health and wellbeing. Fungi have a prolonged and close relationship with human beings, especially at the chemical level. Particularly, they have drawn great interest for their capacity to biosynthesize a variety of structurally unique metabolites that possess promising bioactivities [1,2,3,4,5]. Additionally, the current advances in genetics, synthetic biology, natural product chemistry, and bioinformatics have considerably reinforced the capability to mine their genomes for discovering novel drugs [6].

The genus *Aspergillus* (Aspergillaceae) is one of the most widespread, diversified genera, comprising 400 filamentous species of substantial pharmaceutical, biotechnological, and commercial values [7,8,9,10]. Some of its species have promising enzyme production capacity, as well as causing various illnesses in humans and animals [7,11,12]. They cause various clinical infections that range from allergic and chronic infections to acute invasive aspergillosis [13]. On the other hand, its species are renowned, prolific producers of various metabolites, such as butyrolactones, polyketides, xanthones, sterols, anthraquinones, terpenoids, peptides, and alkaloids, which demonstrate various bioactivities [7,14,15].

*Aspergillus ochraceus* Wilh is a widely distributed fungus that was isolated from various sources, such as decaying vegetation, soils, a variety of agricultural commodities, and moldy grains [16]. The fungus was also known as an important food pathogen that is responsible for the production of carcinogenic mycotoxins, such as ochratoxins, penicillic acid, dihydropenicillic acid, and viomellein [17]. The toxicological importance of this fungus is highlighted by a disease known as “Balkan nephropathy”, which has been linked to the consumption of food products contaminated with penicillic acid and ochratoxin A (**14**) [17,18,19]. On the other hand, this fungus is capable of the biosynthesis of other metabolites, such as isocoumarins, pyrazines, sterols, indole alkaloids, diketopiperazines, polyketides, peptides, quinones, benzodiazepines, and nitro-benzoyl sesquiterpenoids [16,20,21,22,23,24,25,26,27,28,29,30,31,32,33,34,35]. Most of them demonstrate promising bioactivities, that are antimicrobial, cytotoxic, antiviral, anti-inflammatory, antioxidant, anti-Parkinson’s disease, insecticidal, and neuroprotective [16,20,21,22,23,24,25,26,27,28,29,30,31,32,33,34,35]. Due to their intriguing structural features and remarkable diverse bioactivities, they have become a fascinating target for biosynthesis, chemical synthesis, and bioactivity investigations. Therefore, this work aimed at comprehensively exploring the diverse metabolites separated and characterized from *A. ochraceus* associated with different sources regarding their biosynthesis and bioactivities, which were categorized here according to their chemical classes. Additionally, the enzymes produced by this fungus and their possible applications have been discussed [36,37,38,39,40,41,42]. The reported data including the metabolites, sources, and activities have been illustrated. This work could provide the researchers and readers with an overview of the uninvestigated perspectives of *A. ochraceus* as a producer of novel biometabolites. The collected data were obtained via searching through different databases, such as Science-Direct, Web of Knowledge, Scopus, Wiley Online Library, Taylor & Francis, JACS, PubMed, Google Scholar, and Springer.

## 2. Enzymes of *A. ochraceus* and Their Applications

Fungi are fascinating producers of various enzymes that have beneficial contributions in the industrial field. *A. ochraceus* produces a variety of enzymes, which are reviewed in this work, along with their possible biotechnological and industrial values.

### 2.1. Hydrolases

#### 2.1.1. Glycoside Hydrolases

Lignocellulosic biomass is one of the alternative energy sources to fossil fuel that is composed of hemicelluloses, cellulose, and lignin [4,5,43,44]. Cellulases are the principal catalytic enzymes for lignocellulosic biomass hydrolysis, involving β-D-glucosidase, cellobiohydrolase, and endoglucanase, that synergistically hydrolyze cellulose into glucose [4,5,43,44]. They are applicable in the fermentation industry, which needs stability under extreme bioprocessing conditions and high yield. Coir pith or coconut pith is a byproduct of the coir industry with 25% cellulose that is possibly utilized as substrate for saccharification.

Asha et al. purified and characterized β-glucosidase (AS-HT-CeluzB) and processive-type endoglucanase (AS-HT-CeluzA) from *A. ochraceus* MTCC1810, which bio-converted delignified coir pith to glucose for subsequent bioethanol production [37]. These enzymes possessed optimal total cellulase (28.15 FPU/mL), endoglucanase (35.63 U/mL), and β-glucosidase (15.19 U/mL) capacities at pH 6/40 °C. Accordingly, these enzymes could be utilized as synergistic cellulases for complete cellulose saccharification in bio-refineries [37].

Xylan is a major component of the plant cell wall. It consists of 1,4-connected β-D-xylopyranose residues [4,5,43,44]. Xylanases facilitate xylan hydrolysis, primarily utilized in the kraft operation for removing the generated LCC (lignin–carbohydrate complex), which is a physical barrier towards bleaching chemicals entry [45]. Chemical bleaching relies on the utilization of a large quantity of chlorine and chlorine-related chemicals that results in bio-accumulating, mutagenic, toxic, and bio-harmful byproducts [46]. Alternatively, xylanases used in the paper and pulp industry is an eco-friendly method that minimizes pulp fibers’ damage and generates superior quality dissolving pulps [47]. The production of microbial xylanases using various lignocellulosic residues as growth substrates have received great attention because of its low cost and high yield [45,46,47].

Betini et al. reported the production of xylanases from *A. ochraceus* under SSF (solid-state fermentation) using agro-industrial residues (e.g., wheat bran, rice straw, oatmeal, corncob, and *Eucalyptus grandis* sawdust) [39]. It was found that xylanase production (20%) was favored when a mixture of corncob and wheat bran was utilized. The bio-bleaching assay of these enzymes revealed twice to thrice times increased brightness and maintained viscosity, indicating that their use could assist the reduction of chlorine compound concentration in cellulose pulp treatment [39]. In another study, *A. ochraceus* produced high levels of cellulase-free xylanase in oat spelt or birchwood xylan media using wheat bran residue. The enzyme had maximal activity at 65 °C and 5.0 pH [48]. It caused the bleaching of eucalyptus kraft pulp. The results could improve the economic characteristics of bio-bleaching technology and minimize the pollutant compounds used in the process [48]. In 2012, Michelin et al. studied the production of xylanase by *A. ochraceus* utilizing wheat straw autohydrolysis liquor as a carbon source [49]. It was found that the best yield of β-xylosidase and xylanase was obtained when *A. ochraceus* was cultivated with 1% wheat bran added to 10% wheat straw liquor in a stirred tank bioreactor, suggesting the possibility of scaling up this process for commercial production [49]. The enhancement of xylanase and p-xylosidase productivity by *A. ochraceus* using various chemical and physical mutagenesis was assessed [40]. It was found that the NG-13 (N-methyl-N′-nitro-N-nitrosoguanidine) mutant strain secreted high levels of β-xylosidase and xylanase during growth on agricultural waste and commercial xylan, which were stable with optimal activity at pH 5–10 and temperature 45–50 °C [40].

Invertases (β-D-fructo-furanosidases) hydrolyze polysaccharides and sucrose to produce glucose and fructose [50]. The resulting glucose and fructose mixture is called inverted sugar. Invertases are substantial in the food industry, particularly in confectionery for artificial sweetener preparation and increasing sweetening properties [50].

Ghosh et al. (2001) purified invertase enzymes from *A. ochraceus* that was thermotolerant with high sucrose and raffinose affinity [41]. *A. ochraceus* also produced high levels of an extracellular thermostable β-D-fructofuranosidase using sugar cane bagasse-supplemented Khanna medium at 40 °C [51]. This enzyme had a hydrolytic activity with no trans-fructosylating potential. Furthermore, it was positively affected by glucose, which distinguished it from the other β-d-fructofuranosidases, supporting its application for fructose syrup production and sucrose hydrolysis [51].

#### 2.1.2. Proteolytic Enzymes

Medical interest has been drawn to the thrombolytic enzymes of microbial origin. These enzymes act directly by dissolving blood clots as plasmin or as blood plasminogen tissue activators [52]. Protein C prevents blood hyper-clotting in hemostasis [53]. Protein C activator’s addition to the blood inactivates clotting factors VIII and V, which are necessary for thrombin formation, resulting in the prolongation of the partial thromboplastin time [54]. Discovering protein C activators from microbes could be beneficial for clinical practice use because of the low cost.

From *A. ochraceus* 513, proteinase belonging to protein C activator types was separated and assessed for anti-coagulant and fibrinolytic potential [38]. This enzyme was efficient as *Agkistrodon* snake venom protein C activator in thrombin formation time prolongation [38]. Osmolovskiy et al. stated that extracellular proteinases produced by submerged cultures of a micromycete *A. ochraceus* L-1 exhibited specific fibrinogenolytic and fibrinolytic potential, whereas the highest effectiveness was observed at pH 7.0 and 28 °C [55].

#### 2.1.3. Tannases

Tannases (tannin acyl hydrolase) catalyze the hydrolysis of depside and the ester bonds of hydrolyzable tannins [56]. They have been utilized as clarifying agents in the industrial processing of coffee-flavored soft drinks and fruit juices, instant teas manufacture, gallic acid production, and treatment of polyphenolics-contaminated wastewaters, as well as the removal of tannin from foodstuffs and animal feeds [57,58].

*A. ochraceus* was reported to yield extracellular thermostable tannase with distinctive monomeric structural characteristics. This enzyme was activated by manganese, revealing its biotechnological potential for gallic acid production [42]. Furthermore, it was found that *A. ochraceus* biofilm produced tannase in Khanna medium containing tannic acid (1.5% *w*/*v*, carbon source), which was higher than that obtained using conventional submerged fermentation. This enzyme exhibited potent effectiveness at pH 6.0 and 30 °C and was not affected by detergent and surfactant addition [36]. It had different biotechnological applications in propyl gallate production, tannin-rich leather effluent treatment, and sorghum feed formulation [36]. Thus, fungal biofilm is an interesting alternative to produce high levels of tannase with the biotechnological potential to be applied in different industrial sectors.

### 2.2. Oxidases

Alcohol oxidases catalyze the oxidation of alcohols to the corresponding carbonyl compounds with a concomitant release of hydrogen peroxide [59]. They have potential applications in biosensors and the biocatalytic production of different carbonyl compounds that are beneficial in pharmaceutical, flavor, and clinical industries [59]. *A. ochraceus* AIU031 secreted alcohol oxidase (AOD), belonging to the same group as methylotrophic yeast AOD. It oxidized short-chain primary alcohols and ethylene glycol [60]. It was found to possess an optimal ethanol oxidation capacity at 50–55 °C and pH 5–7 [60].

## 3. Applications of *A. ochraceus*

### 3.1. Bioethanol Production

Bioethanol is a liquid biofuel that could be produced from various biomass resources (corn, wheat, paddy straw, sugar beet, wood, municipal waste, forestry byproducts, etc.) through microbial fermentation [61]. This process provides an economically competitive source of energy for biofuel production from cellulosic and lignocellulosic materials.

*A. ochraceus* cellulase demonstrated high hydrolyzing potential of sawdust and its ethanol yield in shaking fermentation than in stationary fermentation. The results supported the use of sawdust as a potential substrate for bioethanol production [62].

### 3.2. Dye Decolorization

Kadam et al. developed a consortium of *A. ochraceus* NCIM-1146 and *Pseudomonas* sp. SUK1 to decolorize adsorbed dyes from textile effluent wastewater under solid-state fermentation [63]. This consortium had a remarkable potential to decolorize adsorbed textile dyes from CPTDE (chemical precipitate of textile dye effluent) and textile wastewater on rice bran. This influence was strongly referred to as the synergism of the excreted extracellular enzymes, such as laccase, azoreductase, NADH-DCIP reductase, and tyrosinase [63]. This approach could be effective in reducing effluent volume through adsorbing textile dye on available agricultural waste residues at a low cost, as well as its disposal or bioremediation to nontoxicity by solid-state fermentation [63]. Saratale et al. reported that *A. ochraceus* remarkably decolorized cotton blue and malachite green dyes, while it had less decolorization capacity on methyl violet and crystal violet [64]. This effect was found to be due to microbial metabolism, not biosorption. Therefore, *A. ochraceus* could be used for bioremediation of cotton blue and malachite green dye-containing wastewater [64]. In addition, the intracellular blue laccase produced by *A. ochraceus* NCIM1146 had maximum substrate specificity toward ABTS (2,2′-azinobis, 3-ethylbenzothiazoline-6-sulfonic acid) and decolorized azo dyes with an optimal effect at 60 °C and 4.0 pH [65].

### 3.3. Kerosene Biodegradation

Accidental spillage of petroleum and its byproducts represents an environmental problem as it causes acute toxicity in some forms of aquatic life [66]. Fungi and bacteria are known for their capacity for petroleum hydrocarbon consumption [67]. Due to their degrading potential, they can be a promising alternative for reducing the environmental impacts of oil spills.

*A. ochraceus* was found to possess kerosene-degrading potential when the previously grown mycelium was incubated in kerosene-containing broth due to its alkane-oxidizing capacity [68]. It also revealed high levels of kerosene biodegradation, aminopyrine N-demethylase, and NADPH-DCIP reductase. The production of acetaldehyde could be utilized as an indicator for kerosene biodegradation [68]. In another study, *A. ochraceus* also showed a noticeable ability to utilize and degrade gasoline and crude oil [69].

### 3.4. Nanoparticles (NPs) of A. ochraceus

Intracellular silver nanoparticles synthesized by exposure of Ag^+^ ions to *A. ochraceus* were heat-treated in an N_2_ environment to yield AgNPs embedded in carbonaceous supports. These carbonaceous AgNPs had an antibacterial capacity versus *B. subtilis* and *E. coli*, and an antiviral potential towards the M-13 phage virus [70].

## 4. Secondary Metabolites from *A. ochraceus* and Their Bioactivities

### 4.1. Isocoumarin Derivatives

**Figure 1:**
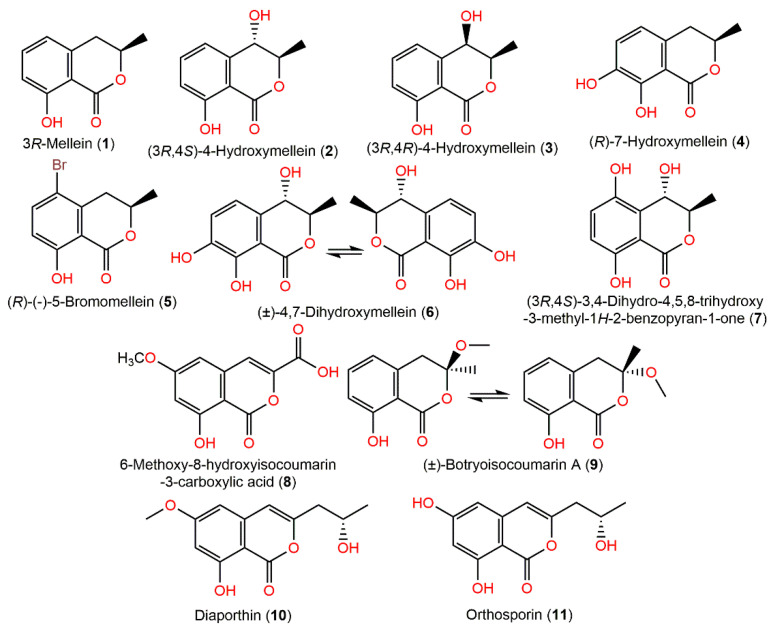
Isocoumarin derivatives (**1**–**11**) reported from *A. ochraceus*.

Dai et al. stated that **1** exhibited anti-HCV potential by prohibiting HCV protease (IC_50_ 35 µM) [20]. Compounds **2**–**4** and **9** were separated from a marine mangrove *Bruguiera gymnorrhiza* rhizospheric soil-associated strain by Liu et al. [71]. The X-ray of **9** proved its racemic nature. They had no potential in the brine shrimp assay; however **4** displayed a selectivity towards *V. harveyi* with MIC 8 μg/mL as that of chloramphenicol [71]. Chromatographic investigation of the EtOAc fraction of *A. ochraceus* MCCC3A00521 resulted in a new pair of enantiomers (±)-4,7-dihydroxymellein (**6**), together with (3*R*,4S)-4-hydroxymellein (**2**) by SiO_2_, RP-18 CC, and HPLC. Their configurations: 3R/4S and 3S/4R for **6** and 3R/4S for **2** were established by NMR, ECD, and XRD (X-ray diffraction) analyses [21]. These metabolites were evaluated for their antioxidant capacity in ABTS, DPPH, and FRAP assays and cytotoxic activity versus SH-SY5Y cells in CCK-8, as well as neuroprotective effectiveness by measuring the GSH level using ELISA assay. The antioxidant results showed that **6** revealed more effectiveness (IC_50_ 62.9 and 70.92 μM for DPPH and ABTS, respectively) than BHT (DPPH, IC_50_ 91.35 μM) and Trolox (ABTS, IC_50_ 101.23 μM). Moreover, **6** had promising cytoprotective potential on H_2_O_2_-induced oxidative damage in SHSY5Y cells without cytotoxic effect. Furthermore, it exerted its cytoprotection efficacy via the GSH level upregulation and ROS elimination, thus protecting SH-SY5Y cells from H_2_O_2_ damage and suggesting its protective role on neurodegenerative illnesses with oxidative stress [21]. Interestingly, NaBr addition to *Chondria crassicualis*-associated *A. ochraceus* fermentation culture induced the production of a new mellein-brominated analog, (R)-(–)-5-bromomellein (**5**), along with (3R)-mellein (**1**) (Figure 1). They possessed antioxidant potential (IC_50_ 24 and 25 μM, respectively) compared to l-ascorbic acid (IC_50_, 20.0 μM) [72]. Yamazaki et al. purified and characterized **8** colorless needles by crystallization from MeOH and elucidated by spectral analysis [73].

**Figure 2:**
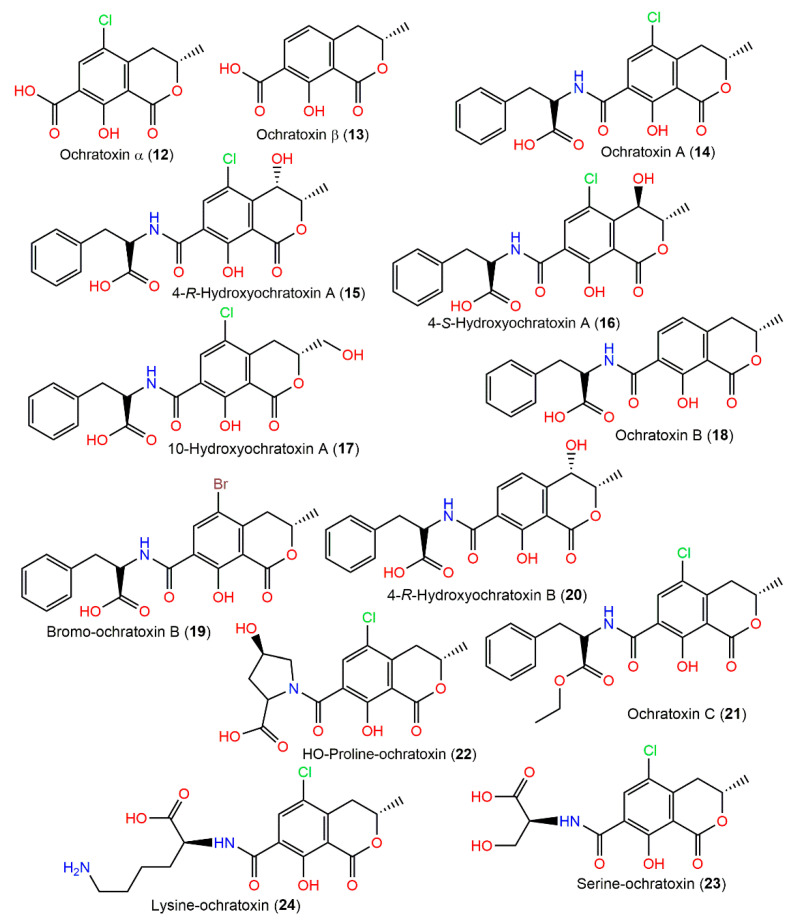
Isocoumarin derivatives (**12**–**24**) reported from *A. ochraceus*.

Ochratoxins are toxic metabolites, biosynthesized by this fungus, that contaminate a variety of foodstuffs, resulting in serious effects on both animals and humans. Ochratoxin A (**14**) is the highly toxic one of this group. It is implicated in nephrotoxic syndromes in various animals, as well as its hepatotoxic, carcinogenic, genotoxic, immune-toxic, and teratogenic effects on animal species and potent carcinogenic effects on humans. It is worth mentioning that ochratoxins A (**14**) and B (**18**) are well-known mycotoxins that are associated with diverse animal and human diseases, including porcine nephropathy, poultry ochratoxicosis, and human endemic nephropathies [17]. Ochratoxin A (**14**) features 7-carboxy-5-chloro-8-hydroxy-3,4-dihydro-3R-methyl isocoumarin, connected by an amide bond to L-β-phenylalanine at 7-carboxy group and, together with its dechloro and ethyl ester derivatives (ochratoxins B (**18**) and C (**12**), respectively) these were purified by SiO_2_ and ion exchange (Dowex I) CC from the fungal CHCl_3_-MeOH extract and characterized by NMR and chemical method [74] (Figure 2). Moreover, **14** was found as a major metabolite, in addition to **18** in the CHCl_3_ extract of *A. ochraceus* NRRL-3174 [75]. In 1971, Cole et al. reported the separation of a new metabolite, 4-hydroxymellein (**2**), along with **1** from the CHCl_3_ extract utilizing SiO_2_ CC, pTLC (acetone/CHCl_3_ 7:93), and crystallization (CHCl_3_:hexane) [16]. Compound **18** was reported to show strong cytotoxicity versus A2780 (IC_50_ 3.0 μM) compared to cisplatin (IC_50_ 2.2 µM) [17]. The substitution of proton in **18** by chlorine in **14** decreased its cytotoxic effect versus A2780 cells [17].

In 1995, ochratoxins **12**, **13**, **15**, **17**, and **20** were purified from *A. ochraceus* NRRL3174 culture and structurally elucidated by HPLC, MS, and NMR (Table 1) [76].

### 4.2. Pyrazine Derivatives

These metabolites possess pyrazin-2(1H)-one core that are biosynthesized from two amino acid molecules, such as isoleucine, leucine, norvaline, and valine. These compounds were reported from bacteria and fungi and revealed cytotoxic, antibacterial, and brine shrimp toxicity capacities.

**Figure 3:**
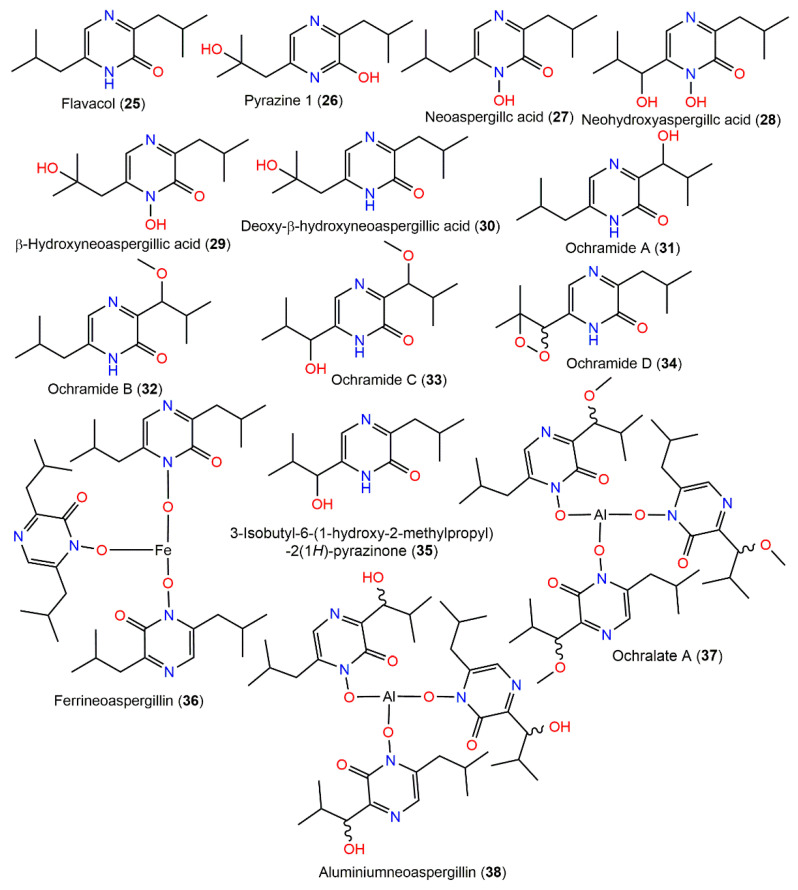
Pyrazine derivatives (**25**–**38**) reported from *A. ochraceus*.

Compound **26**, **25**, and **27** were separated from fungus EtOAc extract by SiO_2_ CC. Compound **26** is structurally similar to **25** with the existence of a β-hydroxyl group in the isobutyl side chain [84] (Figure 3).

Additionally, from the moldy rice-associated strain, neoaspergillic acid (**27**) and five related metabolites (**25**, **28**, **29**, **30**, and **36**) derived from leucine were characterized. Ferrineoaspergillin (**36**) is a red pigment consisting of a complex of iron with neoaspergillic acid [85]. Compound **25** was reported to inhibit NADH oxidase activity with IC_50_ 13 μM by López-Gresa et al. [86]. From the fermentation broth of *A. ochraceus* LCJ11/102 cultured on a 10% NaI-containing nutrient-limited medium, five new pyrazine derivatives, ochramides A–D (**31**–**34**) and ochralate A (**37**), in addition to the formerly reported **25**, **35**, and **38**, were isolated utilizing Sephadex LH-20 and Rp-18 CC and HPLC, and their structures were determined using NMR and X-ray data. Compounds **31**–**33** are 7-hydroxy, 7-methoxy, and 7-methoxy and 11-OH derivative of **25**, respectively, while **34** possesses peroxide linkage among C11 and C12 and **37** is similar to **38** with an additional 7-methoxy group. Compounds **32**, **37**, and **38** possess antibacterial potential towards *E. aerogenes* (MICs 40, 18.9, and 20.1 μM, respectively) compared to ciprofloxacin lactate (MIC 0.93 μM); however, they had no effectiveness versus K562, A549, and Hela cells in the SRB method [22].

### 4.3. Diketopiperazines

Diverse prenylated alkaloids, such as brevianamides, sclerotiamides, stephacidins, and notoamides, which possess bicyclo-[2,2,2]diazaoctane ring or diketopiperazine core, have been separated from *A. ochraceus*. These alkaloids are biosynthetically derived from a second cyclic amino acid residue, L-tryptophan, and two or one isoprene units.

**Figure 4:**
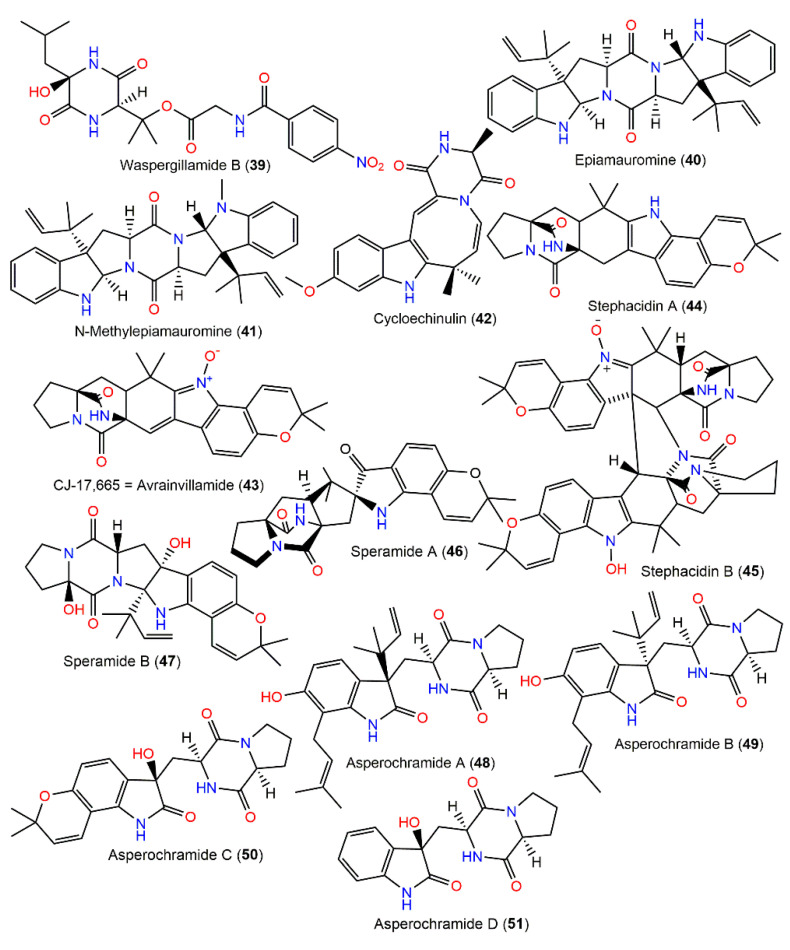
Diketopiperazines derivatives (**39**–**51**) reported from *A. ochraceus*.

Using the OSMAC technique through cultivating Mediterranean sponge *Agelas-oroides*-derived *A. ochraceus* on white beans yielded a new diketopiperazine, waspergillamide B (**39**), featuring an uncommon *p*-nitrobenzoic acid unit and diketopiperazine moiety that was originated from 2-hydroxy leucine and 3-hydroxy valine residues (Figure 4). It was characterized by NMR and its 9R configuration was established by Marfey’s analysis [17].

Three new diketopiperazine derivatives, epiamauromine (**40**), N-methylepiamauromine (**41**), and cycloechinulin (**42**), were obtained from *A. ochraceus* NRRL-3519 sclerotia using Sephadex LH-20 and reversed-phase HPLC and assigned using NMR, whereas their stereochemistry was assured by relying on Marfey’s experiment and alpha D (Figure 4). Compounds **40** and **41** are similar to amauromine obtained from *Amuroascus* sp. [110], possessing dihydroindole and N-methyl dihydroindole moieties, respectively, whereas **42** features an eight-membered ring and indole moiety. These metabolites revealed moderate weight gain reduction on *Helicoverpa zea* (corn earworm) with 30, 17, and 33% reductions, respectively, relative to controls after one week [28]. Compound **42**, a non-basic Trp–Ala-derived alkaloid was separated from *A. ochraceus* D2306 mycelia CHCl_3_ extract by pTLC and characterized by X-ray analysis [87]. A new antibiotic, CJ-17,665 (avrainvillamide, **43**), was isolated from the fermentation broth of soil-associated *A. ochraceus* CL41582 by Sephadex LH-20 CC/HPLC and characterized by NMR tools [88]. This compound is a diketopiperazine metabolite, possessing indole N-oxide moiety. It demonstrated antibacterial capacity versus multi-drug-resistant *S. pyogenes*, *S. aureus*, and *E. faecalis* (MICs 12.5, 12.5, and 25 μg/mL, respectively) in microdilution assay and cytotoxicity towards HeLa cell (IC_90_ 1.1 μg/mL) in the MTT assay [88] (Table 2).

In 2002, new alkaloids, stephacidins A (**44**) and B (**45**), in addition to **43**, were purified from *A. ochraceus* WC76466 EtOAc fraction by Sephadex LH-20 and RP-HPLC and established by NMR and X-ray. They demonstrated cytotoxic effectiveness versus LNCaP, PC-3, A2780/DDP, A2780, A2780/Tax, HCT116/mdr+, HCT-116, MCF-7, HCT116/topo, SKBR3, and LX-1 (IC_50_ ranged from 13.1 to 1.0 µM for **44** and 0.46 to 0.06 µM for **45**), with observed selectivity towards LNCaP cells (IC_50_ 0.06 and 1.0 µM, respectively) [89]. Compound **44** was found to have mild mitochondrial respiratory inhibition capacity (IC_50_ 34 μM) by inhibiting NADH oxidase activity [86].

**Scheme 1:**
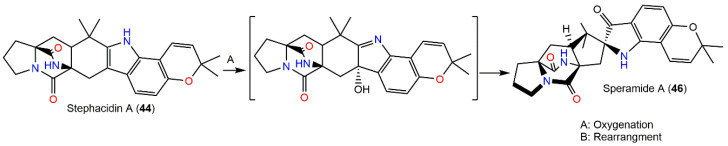
Biosynthesis pathway of speramide A (**46**) from (+)-stephacidin A (**44**) via oxidation and rearrangement [23].

In 2016, Chang et al. reported two new prenylated indole alkaloids, speramides A (**46**) and B (**47**) from *A. ochraceus* KM-007 associated with fresh-water, found using various chromatographic and spectral tools. Compound **46** firstly reported prenylated brevianamide derivatives with a heptacyclic framework possessing azaspiro skeleton with a bicyclo[2.2.2]diazaoctane-linked 2,2-dimethyl-2Hfuran moiety connected to a 3-ox-indole unit. Its bicyclo[2.2.2]diazaoctane diketopiperazine core absolute configuration was 2S/11S/17S/21S based on the CD exciton chirality method. Meanwhile, **47** is related to notoamide D with an additional C-17-hydroxyl group, possessing a 2S/3R/11S/17R configuration. They had no marked effectiveness versus DU145, PC-3, and Lncap cell lines in the MTT assay (IC_50_ > 40 µM). On the other hand, only **46** revealed moderate potential versus *P. aeruginosa* (MIC 0.8 µM) in the 2-fold dilution method [23]. Compound **46** was proposed to be biosynthesized from **44** via oxidation and subsequent rearrangement (Scheme 1) [23].

**Figure 5:**
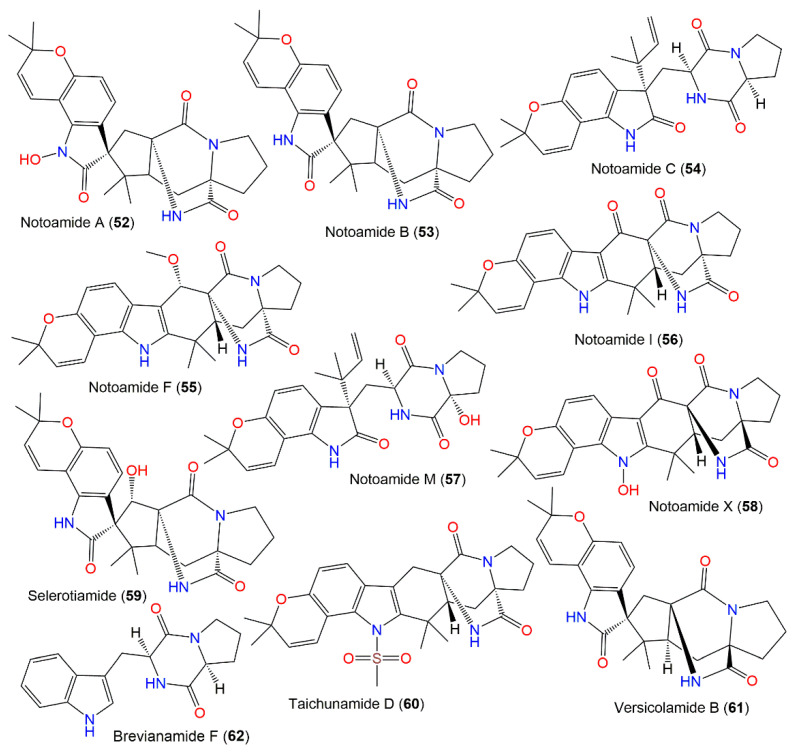
Diketopiperazines derivatives (**52**–**62**) reported from *A. ochraceus*.

New diketopiperazine alkaloids, asperochramides A–D (**48**–**51**), were separated from *A. ochraceus* EtOAc extract, and the formerly reported **44**, **53**, **54**, **57**, **59, 60**, and **62** that were elucidated by spectroscopic, ECD, and X-ray analyses (Figure 5). Compounds **50** and **51** are rare indole diketopiperazine alkaloids with a 3-hydroxyl-2-indolone moiety, while **48** and **49** are epimers at C-3, with 3R/11S/17S and 3S/11S/17S, respectively. These metabolites were assessed for their anti-inflammatory potential using the LPS-stimulated murine macrophage RAW 264.7 cells model [90]. Furthermore, **44**, **48**, **57**, **53**, and **60** prohibited IL-1β (% inhibition ranged from 36.8% to 49.2%, concentration of 20 μM), while others had lower than 30% at a concentration of 20 μM. Meanwhile, **44**, **48**, **53**, **51**, **54**, **59**, **60**, and **62** showed TNF-α inhibition (% inhibition ranged from 32.6% to 41.9%, at a concentration of 20 μM), whereas **50** and **57** exhibited lower TNF-α inhibition ratios than 30% at the same concentration. Hence, **44, 48**, **53**, and **60** revealed potential anti-inflammatory capacity [90]. Furthermore, notoamides A (**52**), B (**53**), I (**56**), M (**57**), and X (**58**) and sclerotiamide (**59**) were characterized from deep-sea fresh-water *A. ochraceus* that had no notable anti-H1N1 virus, anti-food allergic, antimicrobial, and anti-inflammatory capacities [80].

Hu et al. assessed the anti-Parkinson’s disease efficacy of **44**, **53**–**56**, and **61**. All metabolites were tested for their anti-Parkinson’s disease capacity in MPP^+^ (dopaminergic selective neurotoxin)-induced SH-SY5Y cells assay. All metabolites revealed anti-Parkinson’s disease potential (EC_50_s ranged from 2.30 and 7.39 μM), whereas **44** and **56** featured prominent effectiveness (EC_50_ 2.45 and 2.30 μM, respectively) compared to levodopa (EC_50_ 2.06 μM). Their ADMET prediction revealed the appropriate features for CNS drugs, better drug-likeness characteristics, and preferable safety scores of toxicities [24]. Therefore, **56** could be an advantageous lead compound for anti-Parkinson’s therapeutic drugs.

### 4.4. Benzodiazepine Derivatives

Benzodiazepines are psychoactive metabolites that are naturally reported from *Streptomyces*, *Aspergillus*, and *Penicillium* [91,92]. These metabolites are renowned as a chemotaxonomic marker for this fungus [26,86].

**Figure 6:**
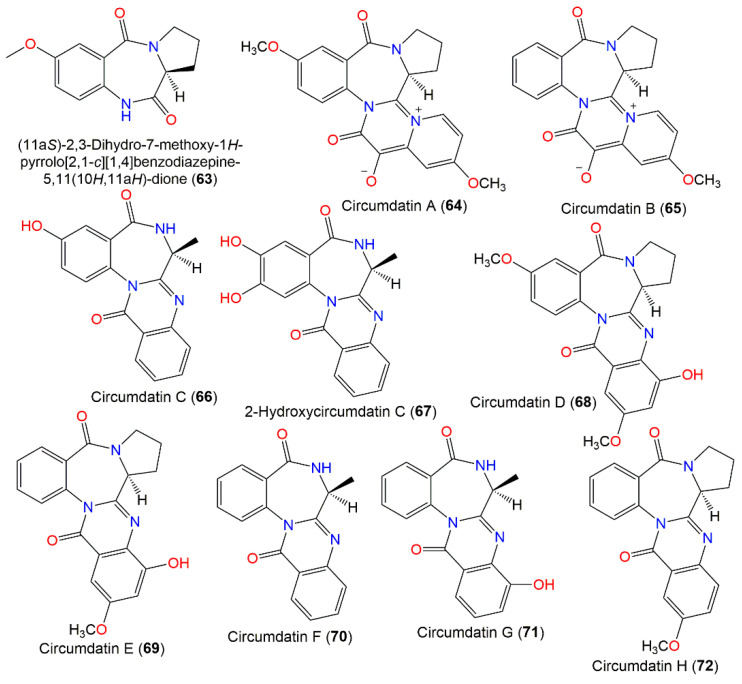
Benzodiazepine derivatives (**63**–**72**) reported from *A. ochraceus*.

New benzodiazepine-related metabolites, compounds **68**–**70**, in addition to the known **64**–**66**, were separated from an *A. ochraceus* terrestrial strain obtained from milosorghum seeds using UV-vis/HPLC analysis and characterized using various spectral analyses (Figure 6). Compound **68** is a C-5 methoxy circumdatin E (**69**), while **70** lacks an OH group at C-5 in circumdatin C (**66**) [92]. The benzodiazepine core of **66** and **70** was formed from L-proline and substituted anthranilic acid, while that of **64**, **65**, **68**, and **69** has L-alanine instead of L-proline [91].

Moreover, a new alkaloid, circumdatin G (**71**) and **66** and **70** were isolated from a sediment-derived strain by Sephadex LH-20 CC and HPLC. Compound **71** is a 15-OH derivative of **70** based on spectroscopic analysis. They exhibited no HCV-NS3 protease inhibition in the scintillation proximity assay (SPA) [20]. In 2005, circumdatin H (**72**), a new derivative from the culture broth of soil-derived *A. ochraceus*, in addition to **69**, was isolated by SiO_2_ CC and HPLC and established by the spectral and chemical method by López-Gresa et al. [86]. Their mitochondrial respiratory chain inhibition capacity was estimated by measuring NADH oxidase potential [86]. These metabolites prohibited (IC_50_ 1.50 and 2.50 μM, respectively) NADH oxidase activity (integrated electron transfer chain), whereas **72** was slightly more potent than **69**, suggesting that the C-15 hydroxy group in **69** might lead to a more difficult interaction with the respiratory chain [86]. Thus, these compounds may act as leads in developing new tools for controlling insects [86]. Compound **64** exhibited antioxidant effectiveness (IC_50_ 32 μM) in comparison to ascorbic acid (IC_50_ 20.0 μM) [72].

The marine brown alga *Sargassum kjellmanianum*-derived *A. ochraceus* produced a new benzodiazepine analog, 2-hydroxycircumdatin C (**67**), as well as the known **66**, **68**, and **70** that were isolated from culture broth and mycelia extract by RP-18, SiO_2_, and Sephadex LH-20 CC and structurally established by spectroscopic and CD tools. Only **67** revealed marked antioxidant potential (IC_50_ 9.9 μM) that was 8.9-fold more powerful than BHT (butylated hydroxytoluene, IC_50_ 88.2 μM); however, **66** and **68** (IC_50_ > 100 μg/mL) had weak potential in the DPPH assay. On the other hand, none of them had antibacterial potential in the well diffusion method [26].

Hu et al. purified a new benzodiazepine derivative, circumdatin N (**75**), in addition to **70** and **71**, from *A. ochraceus* MCCC3A00521 EtOAc extract by SiO_2_, RP-18 CC, and HPLC. Compound **75** is structurally related to **70** except for the existence of an additional C13-OH group. Its 19R configuration was established by ECD and X-ray analyses. They exhibited anti-Parkinson’s effectiveness in MPP^+^ (dopaminergic selective neurotoxin)-induced SH-SY5Y cells assay with EC_50_s 10.77, 5.44, and 7.39 μM, respectively [24].

**Figure 7:**
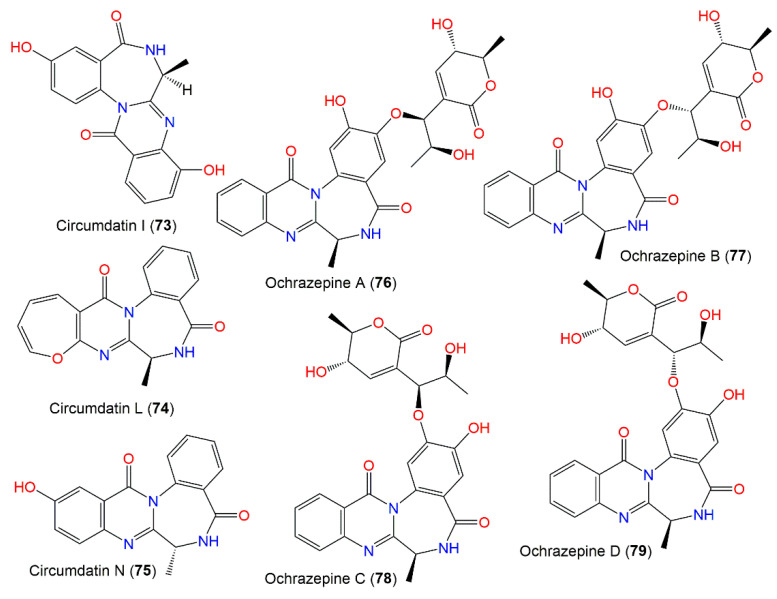
Benzodiazepine derivatives (**73**–**79**) reported from *A. ochraceus*.

**Scheme 2:**
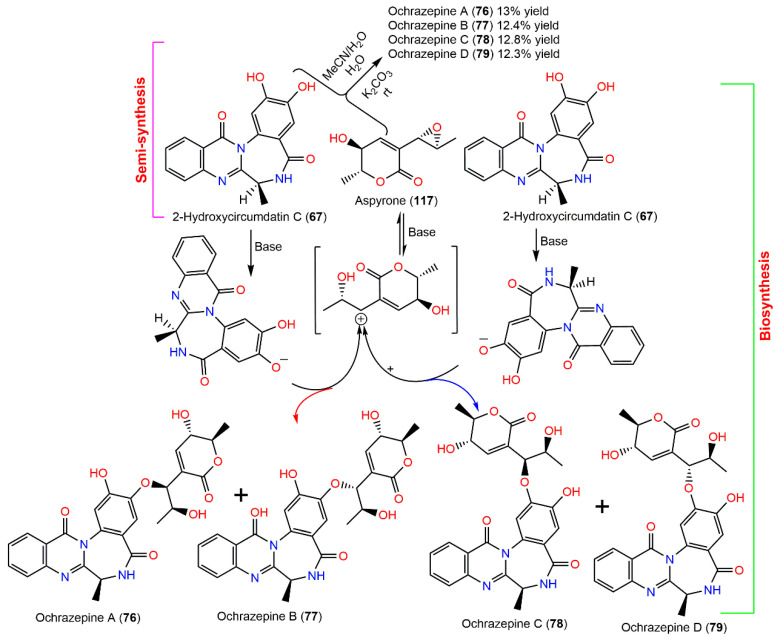
Semisynthetic and biosynthetic pathways of ochrazepines A–D (**76**–**79**) [31].

In 2019, Fan et al. purified four new metabolites: ochrazepines A–D (**76**–**79**) from coral-associated *A. ochraceus* LCJ11102 fermentation broth utilizing SiO_2_, Sephadex LH-20, and HPLC (Figure 7). These metabolites are dimer conjugates derived from **117** and **67** through a nucleophilic addition to epoxide [31] as confirmed by the semisynthesis (Scheme 2).

Their structures and absolute configuration were assured by spectral and chemical methods. These compounds are epimers, differing only in configurations at C-8′. Their cytotoxic potential versus 2 normal and 26 human cancer cell lines was evaluated in the CTG (Cell Titer Glo) assay. Compound **117** had the most broad-spectrum cytotoxic capacity against 16 cancer cells (IC_50_ ranged from 2.54 to 9.79 µM) because the epoxide could alkylate DNA by a nucleophilic addition with the sulfhydryl of protein or the base of DNA. Compound **76** also displayed a wide spectrum cytotoxic potential versus 10 cancer cells (IC_50_ ranged from 3.10 to 11.32 µM). In addition, **67**, **77**, and **79** exhibited a selective cytotoxic capacity versus U251, while **78** selectively prohibited U87, A673, and Hep3B. Interestingly, the conjugation among **67** and **117** promoted the inhibitory potential on U87 and U251. Compounds **76** and **78** with 8′*S* configuration revealed cytotoxic potential versus U87 cells; however, **77** and **79** with 8′*R* configuration were active versus U251 cells. On the other hand, **76**–**79** exhibited low cytotoxicity on the normal human cells, HEK-293F and L02. Thus, these metabolites could represent leads in anticancer drugs against human glioblastoma cells with non-cytotoxic action to human normal cells [31].

### 4.5. Indole and Other Alkaloids

**Figure 8:**
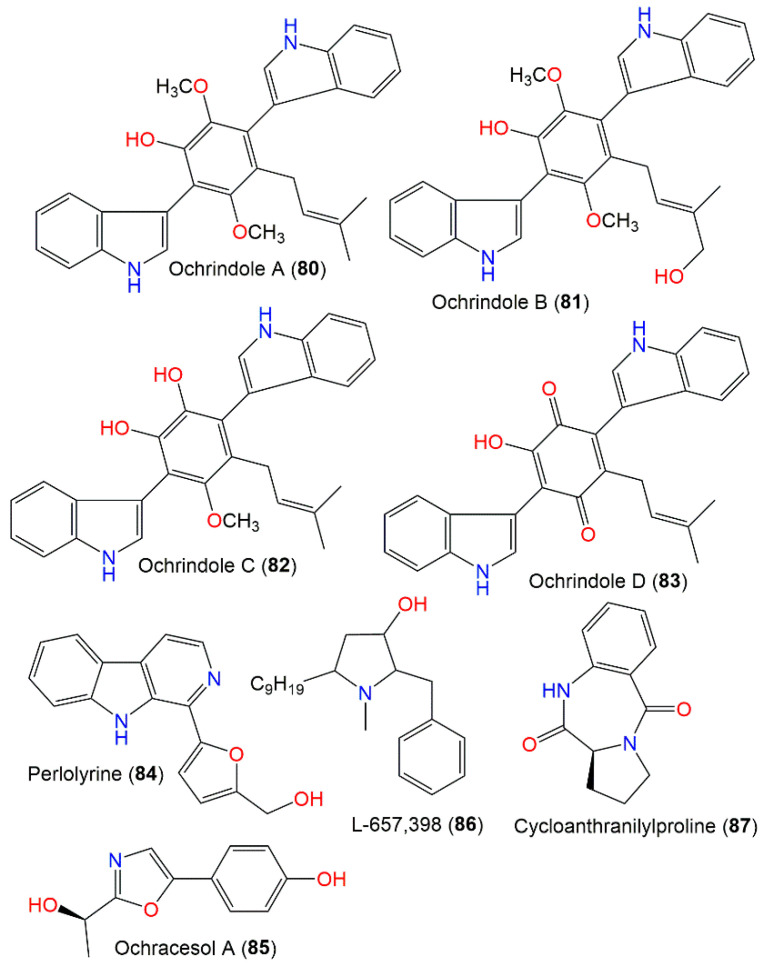
Indole and other alkaloids (**80**–**87**) reported from *A. ochraceus*.

Chromatographic investigation using SiO_2_ and Sephadex LH-20 CC of anti-insectan *A. ochraceus* NRRL3519 sclerotia EtOAc extracts yielded new prenylated bis-indolyl benzenoid metabolites, ochrindoles A–D (**80**–**83**), and a new bis-indolyl quinone, ochrindole D (**83**) (Figure 8). These metabolites display a central ring-linked prenyl sidechain. In feeding assays, they had moderate effectiveness (concentration of 200 ppm *w*/*w*) towards *Carpophilus hemipterus* (dried fruit beetle) and *H. zea*, whereas **80** caused a 20% and 30% weight gain reduction, respectively [29]. These compounds also had potential versus *B. subtilis* (inhibition zone 15–18 mm, concentration of 100 μg/disk) in the disk assay. It is worth mentioning that these compounds were found to be the main components of *A. ochraceus* NRRL3519 sclerotia; therefore, they could be accountable for the anti-insectan potential of the *A. ochraceus* extracts [29]. Perlolyrine (**84**), a β-carboline alkaloid with a 5-hydroxymethyl substituent in the C-1 linked furan ring, was reported from *A. ochraceus* MCCC3A00521 culture by Hu et al. [24]. Furthermore, *A. ochraceus* ATCC22947 yielded a novel pyrrolidine antibiotic, L-657,398 (**86**), that was separated from mycelia EtOAc extract by SiO_2_ and Sephadex LH-20 CC and elucidated by various NMR spectroscopic data. This metabolite is related to anisomycin, an anti-protozoal and antifungal metabolite produced by *Streptomyces roseochromogenes* and *S. griseolus* [111]. Compound **86** also possessed marked broader efficacy towards yeasts and filamentous fungi than anisomycin using the disk diffusion method [93]. Ochracesol A (**85**), a new alkaloid, featured an oxazole ring linked to p-OH-disubstituted benzene. Its 17R configuration was assigned by X-ray and ECD analyses. This compound had weak anti-Parkinson’s potential (EC_50_ 17.84 μM) [24].

### 4.6. Peptides

Aspochracin (**88**), a cyclotripeptide with N-methyl L-valine, N-methyl L-alanine, and L-ornithine residues and an octatrienoic acid side chain, was separated as a pale-yellow powder from *A. ochraceus* EtOAc extract by SiO_2_ CC and characterized using NMR and chemical methods. It was tested for its insecticidal, antimicrobial, and cytotoxic activities. It demonstrated insecticidal potential by injection of silkworm and fall webworm, with 17 μ/g minimal concentration, on larvae, causing paralysis followed by death into the final instar larvae of silkworm and 170 μ/g for the final instar larvae of fall webworm. Additionally, it demonstrated the silkworm’s first instar larva and egg contact toxicity using the dipping method. Its hydrogenation completely minified the effect, suggesting the potential of the side chain’s conjugated triene in its action. Its 165 mg/kg IV dose did not kill mice and had no antimicrobial efficacy towards various microbes in agar dilution, paper disc, and liquid dilution methods [95].

### 4.7. Sesquiterpenoids

**Figure 9:**
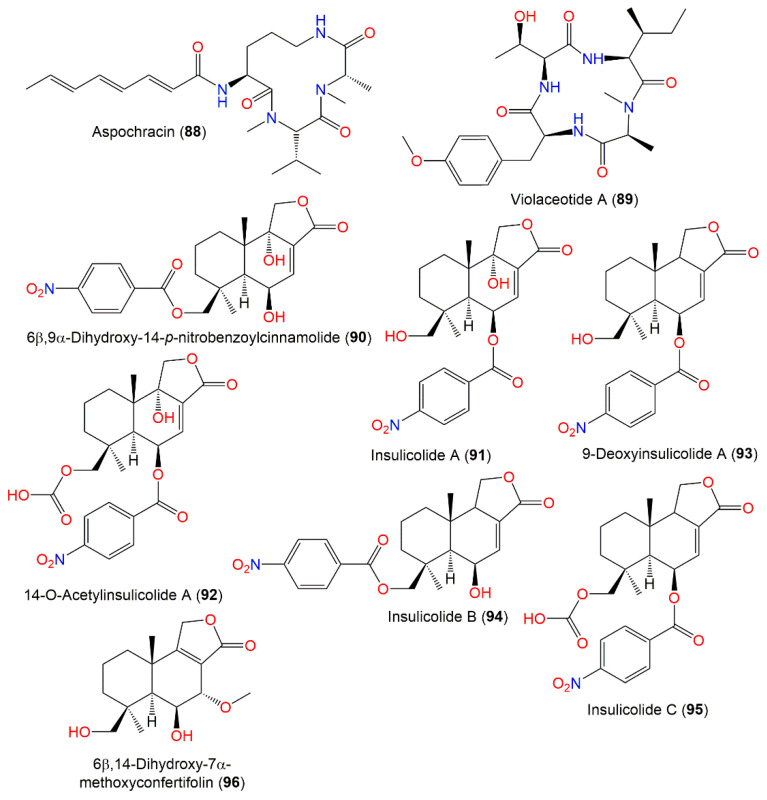
Peptides (**88**–**89**) and sesquiterpenoids (**90**–**96**) reported from *A. ochraceus*.

Nitrobenzoyl sesquiterpenoids are a rare class of metabolites and were reported mainly from *Aspergillus* species [25]. *A. ochraceus* Jcma-1F17 associated with *Coelarthrum* sp. marine alga collected from Paracel Islands, South China Sea yielded a new nitrobenzoyl sesquiterpenoid, 6β,9α-dihydroxy-14-*p*-nitrobenzoylcinnamolide (**90**), and a former analo, **91**, that were separated from mycelia EtOAc extract by SiO_2_ CC and HPLC and characterized by NMR, MS, CD, and optical rotation (Figure 9). They displayed noticeable cytotoxic potential versus HL-60, U937, H1975, MCF-7, BGC-823, Hela, K562, Molt-4, A549, and Huh-7 (IC_50_ ranged from 1.95 μM to 6.35 μM) in the CCK-8 assay [32]. Additionally, **91** displayed marked in vitro effectiveness versus HCT-116 and moderate selectivity towards a panel of renal tumor cell lines [25]. Moreover, **90** had moderate antiviral activity versus H3N2 and EV71 (IC_50_ 17.0 and 9.4 μM, respectively) while **91** had no obvious potential in the CPE (cytopathic effect) and CCK-8 assays. On the other hand, they did not show any obvious effect versus *M. tuberculosis* H37Ra in the GFPMA assay [32]. Compounds **94** and **95**, new nitrobenzoyl sesquiterpenoids, along with **90**–**93** and **96**, were isolated from culture EtOAc extracts of *A. ochraceus* Jcma-1F17 derived from marine alga *Coelarthrum* sp. collected from the South China Sea [97]. Their isolation and elucidation were done using SiO_2_ CC/Sephadex LH-20/HPLC and NMR/MS/ ECD spectra, respectively. Compound **92** was previously synthesized by treating **91** with acetic anhydride in pyridine [112]. These metabolites were assessed for cytotoxic capacity versus OS-RC-2, ACHN, and 786-O cells in the CCK-8 assay, as well as for NF-κB inhibition using LPS-stimulated RAW264.7 [97]. Compound **91** revealed potent effectiveness versus OSRC-2, ACHN, and 786-O cells (IC_50_s 1.5, 1.5, and 0.89 μM, respectively) compared to sorafenib (IC_50_s 7.0, 3.4, and 4.9 μM, respectively), whereas **92** had (IC_50_ 5.3, 4.1, and 2.3 μM) potent potential comparable to sorafenib (Nexavar), the approved drug for advanced renal cell carcinoma treatment, revealing that the C-9-OH group might contribute more cytotoxic capacity towards renal carcinoma cells [97]. Furthermore, **92** induced G0/G1 phase cell cycle arrest and late apoptosis at concentrations of 1 and 2 μM, respectively, after 72 h in 786-O cells. On the other hand, only **94** demonstrated weak inhibition of LPS-induced NF-κB activation (16%, concentration of at 4 μM), while **92** and **94**–**96** displayed weak toxic influence on RAW264.7 cells [97].

### 4.8. Polyketides

**Figure 10:**
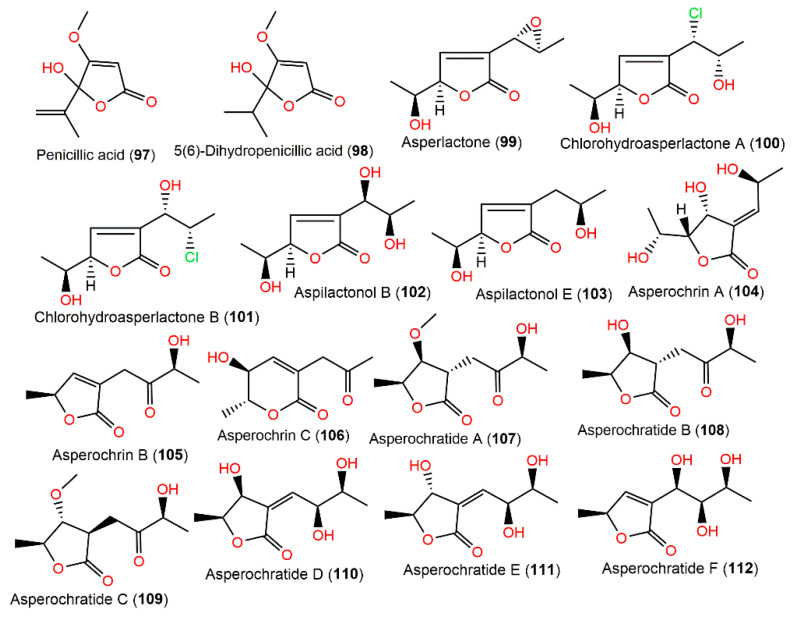
Polyketides (**97**–**112**) reported from *A. ochraceus*.

*A. ochraceus* M#1129-85 EtOAc extract yielded the toxic metabolites penicillic acid (**97**) and 5(6)-dihydropenicillic acid (**98**). It was found that **97** was only produced on potato dextrose agar medium; however, *A. ochraceus* in rice flour yielded **98**, suggesting that **97** was transformed into **98** in high-nutritive circumstances (Figure 10). Interestingly, **98** was not mutagenic in the Ames and *umu* tests [98]. Compounds **99** and **117** were isolated from the mycelia of *A. ochraceus* collected from an apple-packing house environmental sample. It is noteworthy that **117** displayed better antimicrobial influence than **99** versus 22 bacterial strains and 13 fungal strains in the paper disc assay [99].

The investigation of *A. ochraceus* MA15 obtained from marine mangrove *Bruguiera gymnorrhiza* rhizospheric soil resulted in three new metabolites: asperochrins A–C (**104**–**106**), along with the formerly separated **97**, **98**, **100**, **101**, and **118**–**121**. They were elucidated using NMR, CD, and Mosher’s methods. Compounds **104** and **105** featured α,β-unsaturated γ-lactone, while **106** had α,β-unsaturated δ-lactone moiety. They possessed no activity towards *Artemia salina* in the brine-shrimp lethality test. Meanwhile, **97**, **100**, **104**, **118**, and **119** revealed selective antibacterial potential (MIC ranged from 0.5 to 64.0 μg/mL) versus *V. harveyi*, *V. anguillarum*, and *A. hydrophilia*, whereas **97** and **104** were the most powerful metabolites (MICs ranged from 0.5 to 32.0 μg/mL). It was revealed that increased OH groups elevated activity and the terminal C-6(7) double bond (e.g., **97** vs. **98**) was substantial for the activity [71].

**Figure 11:**
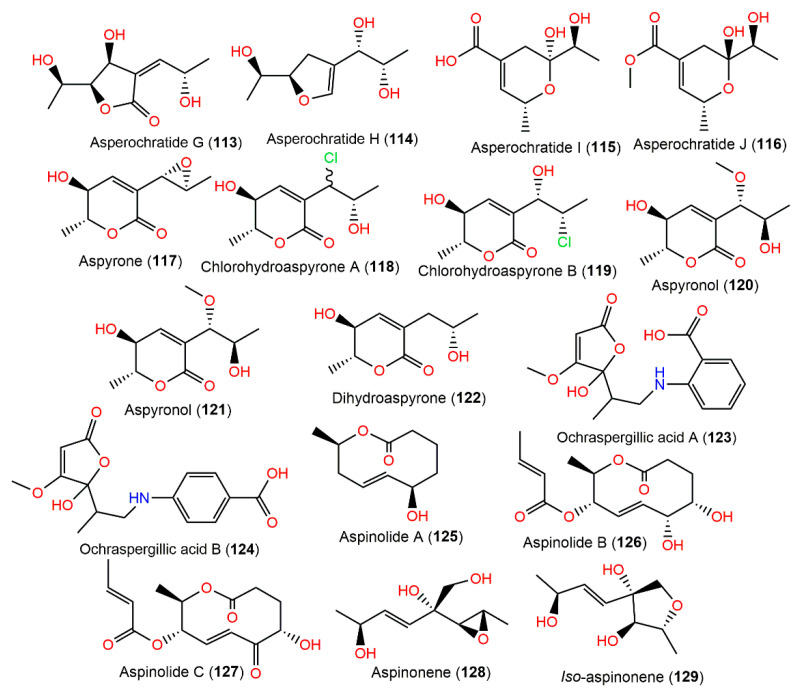
Polyketides (**113**–**129**) reported from *A. ochraceus*.

Co-cultivation of *A. ochraceus* with *Streptomyces lividans* resulted in an increase in the yields of **97** and **98**; however, its co-cultivation with *B. subtilis* yielded two new penicillic acid derivatives, ochraspergillic acids A (**123**) and B (**124**), which are new penicillic acid–aminobenzoic acid hybrids. Interestingly, it was suggested that the aminobenzoic acid moiety of **123** is originated by bacteria [17] (Figure 11).

**Scheme 3:**
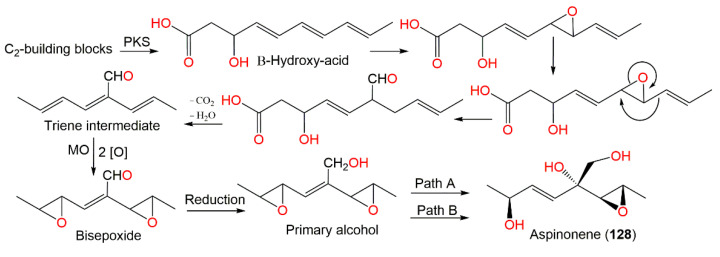
Biosynthesis pathway of aspinonene (**128**) [100]. PKS: Polyketide synthase; MO: monooxygenase; Path A: 1,2-addition + allylic rearrangement; Path B: 1,4-addition.

Aspinonene (**128**), a branched pentaketide with uncommon oxygenation, was separated from *A. ochraceus* DSM-7428 culture broth using SiO_2_ and Sephadex LH-20 CC [100]. Its biosynthesis was proposed utilizing [^18^O_2_]-rich atmosphere fermentation and [^13^C]-labelled acetate feeding experiments (Scheme 3). It was proved that **128** was biosynthesized via the polyketide pathway and post-polyketide modification is a key step that directed aspinonene biosynthesis [100].

New C9 polyketides asperochratides A–J (**107**–**116**) and **102**, **103**, **121**, and **122** were isolated from EtOAc extract of the fermentation broth of deep-sea water-derived *A. ochraceus* using SiO_2_, RP-18, Sephadex LH-20, pTLC, and HPLC and characterized by NMR, MS, Mosher’s method, ICD, ECD, and specific rotation. Structurally, they share the same polyketide skeleton and belong to aspyrone-related metabolites. Compounds **103** and **107**–**111** exerted a significant cytotoxic effect on the BV-2 cell line with inhibitions ranging from 50.29 to 72.81% in the MTT assay. In the anti-inflammation assay, **103** (1 μM) produced a marked decline (44.87%) of NO concentration in the LPS-stimulated BV-2 cells using Griess reagent. However, none of them exhibited significant anti-H1N1 virus, anti-food allergic, and antimicrobial capacities in vitro [80].

### 4.9. Xanthine and Quinone Derivatives and Flavonoids

**Figure 12:**
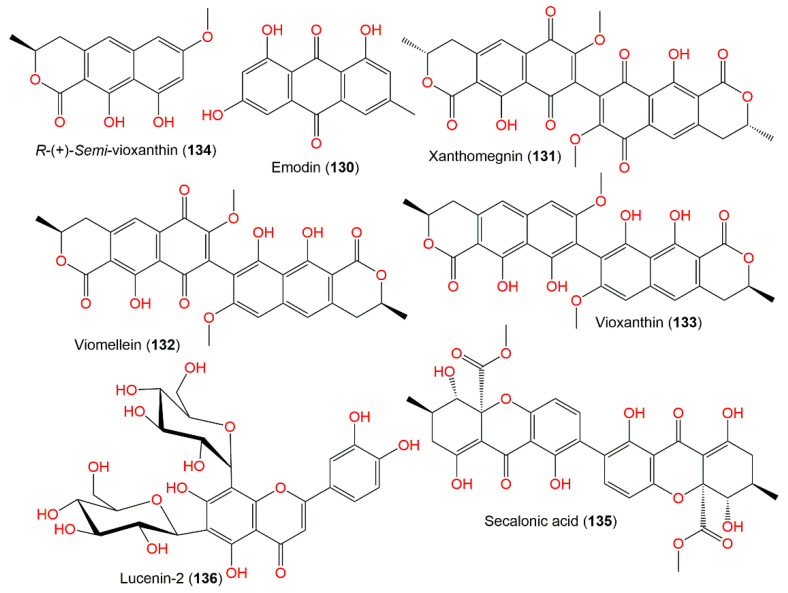
Quinone (**130**–**132**) and xanthine (**133**–**136**) derivatives and flavonoids (**135**) reported from *A. ochraceus*.

Xanthomegnin (**131**) and viomellein (**132**) are structurally related quinones that were produced by the *A. ochraceus* group (Figure 12). These quinones could induce cholangio-hepatitis and hepatic lesions in mice toxicity assay [105], as well as nephrotoxicity in pigs and swine [107] and mycotoxicosis in mice [17].

Additionally, **132** exhibited potent cytotoxic capacity versus A2780 and L5178Y (IC_50_ 5.0 and 5.3 μM, respectively) compared to cisplatin (for A2780, IC_50_ 2.2 µM) and kahalalide F (for L5178Y, IC_50_ 4.3 µM), while it was inactive towards human Jurkat T and Ramos B cell lines. Compound **130** was obtained from *A. ochraceus* isolated from Chinese potato by the solid phase extraction method. The optimal yield of **130** was accomplished when the fermentation was done at pH 7.0 and 32 °C [104]. Secalonic acid A (**135**), a C-4-C-4′ dimer xanthone, was isolated from the EtOAc fraction of *A. ochraceus* Wilh IFM4443. This metabolite was formerly reported from *Claviceps purpurea* [113]. It was not lethal to mice (concentration of 250 mg/kg, orally) and not toxic to the chicken embryos; however, it displayed antibacterial activity towards *B. subtitis* and *piricularia oryzae* in the disc assay [103].

### 4.10. Sterols

**Figure 13:**
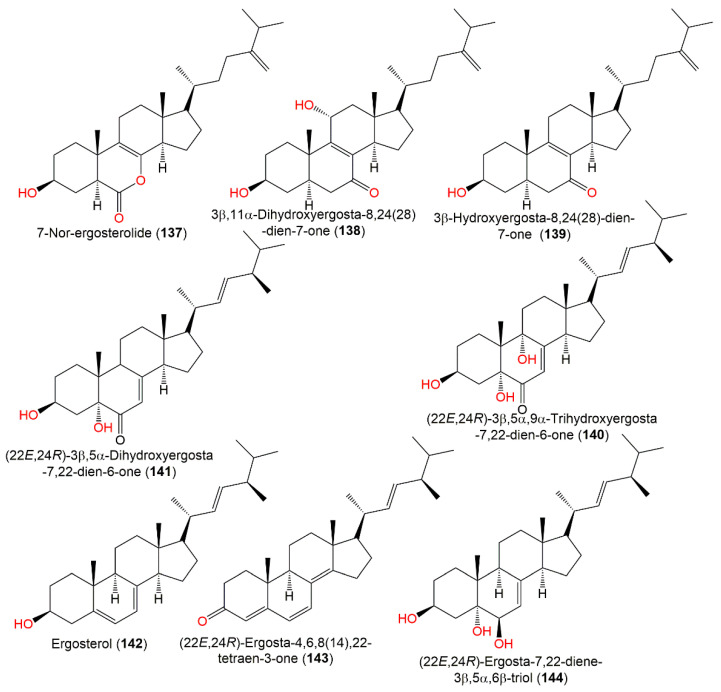
Sterols (**137**–**144**) reported from *A. ochraceus*.

Cui et al. (2010) isolated three new steroids, **137**–**139**, and known steroids, **140**–**148**, from marine brown alga *S. kjellmanianum*-inhabitant *A. ochraceus* EN-31 by SiO_2_ CC. The new compounds were established by spectroscopic analyses and modified Mosher’s method (Figure 13).

**Figure 14:**
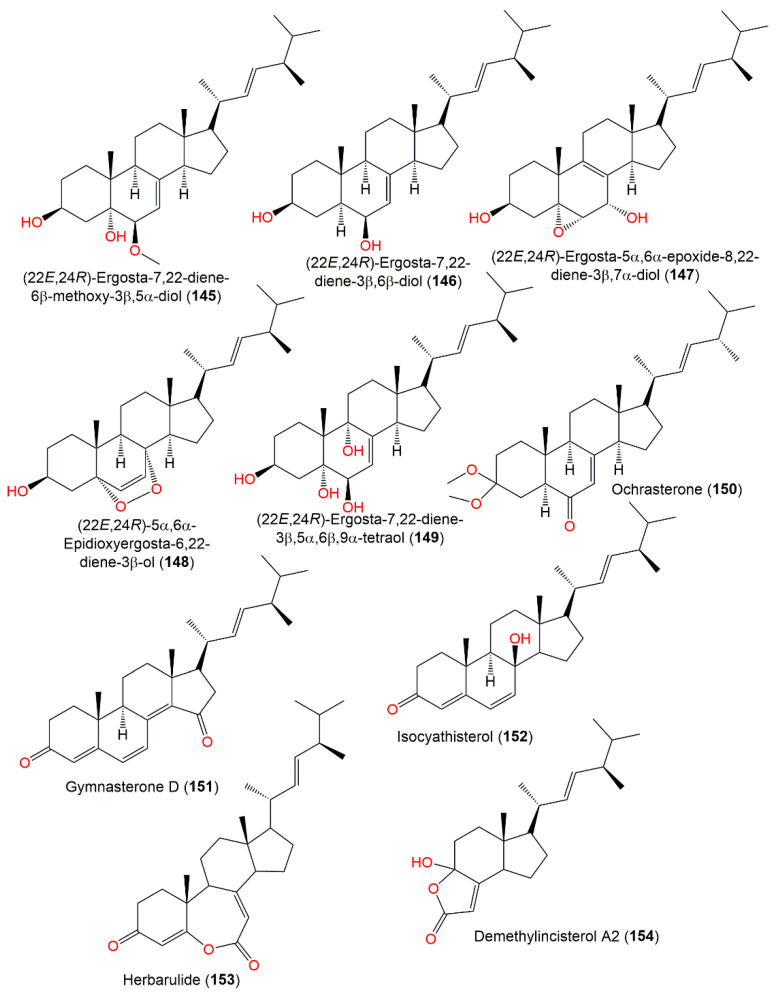
Sterols (**145**–**154**) reported from *A. ochraceus*.

Compound **137** is a rare 7-norsteroid with an uncommon pentalactone B ring system [27]. It demonstrated selective cytotoxic potential towards SW1990, NCI-H460, and SMMC-7721 cell lines (IC_50_ 28.0, 5.0, and 7.0 µg/mL, respectively), while **138** revealed selectivity (IC_50_ 28.0 µg/mL) towards SMMC-7721 in the MTT assay and both had no antimicrobial potential in the agar diffusion test [27]. 22*E*,24*R*-Ergosta-7,22-diene-3*β*,5*α*,6*β*,9*α*-tetraol (**149**), reported from *A. ochraceus* derived from fresh sea water, displayed no notable anti-H1N1 virus, anti-food allergic, and antimicrobial activities [80]. On the other hand, **143**–**154** isolated by Hu et al. from *A. ochraceus* MCCC3A00521EtOAc extract demonstrated anti-Parkinson’s disease efficacy (EC_50_ ranged from 35.71 to 49.53 μM) (Figure 14) [24].

The petroleum ether fraction of *A. ochraceus* MCCC3A00521 afforded a new sterol, ochrasterone (**150**), that was characterized by NMR, ECD, and X-ray. Compound **150** has a 5S/9R/10R/13R/14R/17R/20R/24R configuration and 7-en-6-one motif with dimethoxy-substituted C-3 [21]. It displayed no antioxidant, cytotoxic, or neuroprotective effectiveness [21].

### 4.11. Other Metabolites

**Figure 15:**
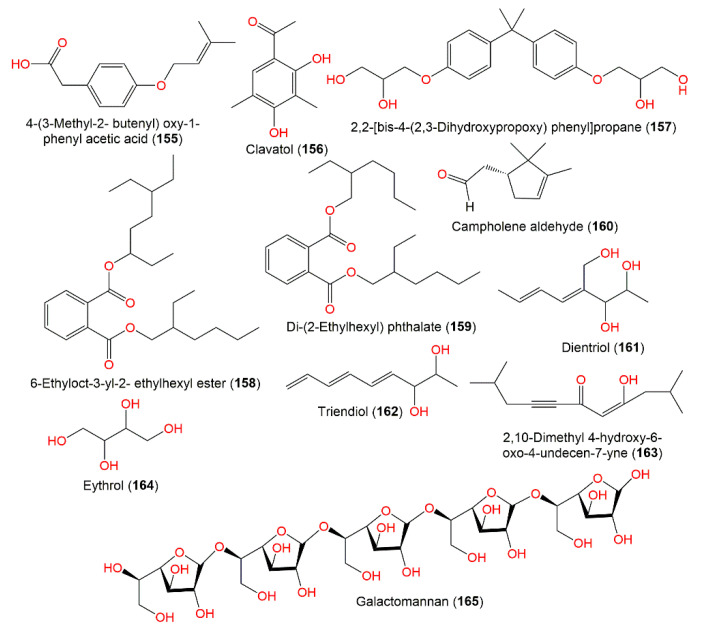
Other metabolites (**155**–**165**) reported from *A. ochraceus*.

The new compounds **155** and **163** were produced by mutant strain *A. ochraceus* NRRL3174 due to UV irradiation of its conidia during the active and stationary growth phases, respectively (Figure 15). They were purified by TLC and HPLC. Compound **155** is a phenylacetic acid derivative. They were tested for antimicrobial potential versus *M. luteus*, *B. subtilis*, *S. aureus*, *M. smegmatis*, *L. monocytogenes*, *K. pneumoniae*, *P. syringae*, and *A. tumefaciens*, *M. ramannianus*, and *S. cerevisiae* in the agar diffusion assay. Compound **163** showed marked effectiveness versus *S. aureus* and *B. subtilis* and no effect on the other strains. However, **155** did not display any effects on any strains [109]. Clavatol (**156**), an acetophenone derivative, had mild antioxidant capacity (IC_50_ 30 μM) in the DPPH assay compared to l-ascorbic acid (IC_50_, 20.0 μM) [72].

Compounds **158** and **160**, purified from sponge-derived *A. ochraceus* MP2, exhibited antimicrobial capacity versus potential pathogens *K. pneumonia* ATCC-15380, *S. aureus* ATCC-25923, and *P. aeruginosa* ATCC-27853 [108].

*Dichotella gemmacea* coral-derived *A. ochraceus* produced AW1 (**165**), a novel galactomannan extracellular polysaccharide, that was characterized using chemical and spectroscopic methods. AW1 could be a marked source for galacto-furanose oligosaccharides and could have food and pharmacology applications [35].

## 5. Conclusions

**Figure 16:**
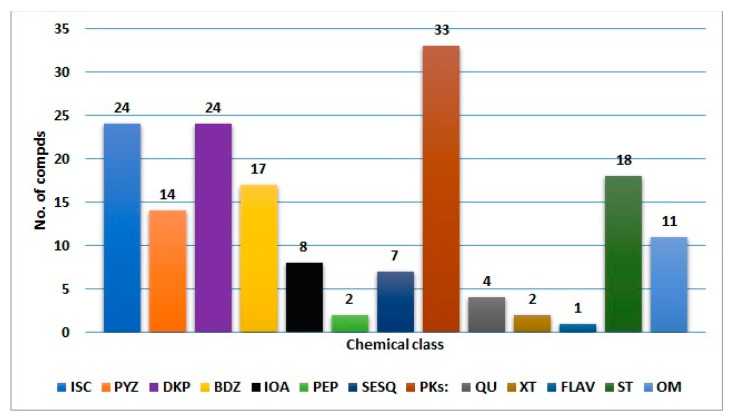
Different metabolites reported from *A. ochraceus*. ISC: Isocoumarins; PYZ: pyrazines; DKP: diketopiperazines; BDZ: benzodiazepine: IOA: indole and other alkaloids; PEP: peptides; SESQ: sesquiterpenoids; PKs: polyketides; QU: quinones; XT: xanthes; FLAV: flavonoids; ST: sterols; OM: other metabolites.

**Figure 17:**
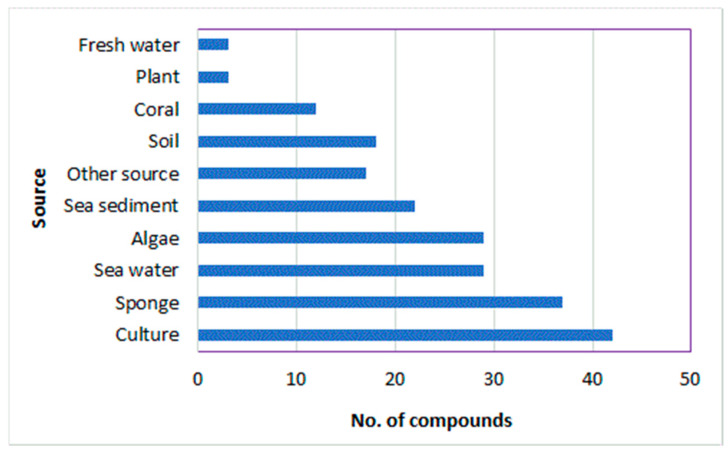
Number of metabolites reported from *A. ochraceus* isolated from various sources.

Fungi have been extensively investigated because of their importance as wealth producers for diverse biometabolites and enzymes, in addition to their industrial, agricultural, and pharmaceutical potential. *A. ochraceus* can biosynthesize diverse biometabolites that may be utilized as leads in the discovery of therapeutic agents for several health concerns. In this review, 165 metabolites belonging to diverse chemical classes (isocoumarins, pyrazines, diketopiperazines, benzodiazepine, indoles, peptides, sesquiterpenoids, polyketides, quinones, xanthines, flavonoids, sterols, and other metabolites) have been reported from *A. ochraceus* isolated from various source in the period from 1965 to June 2022 (Figure 16 and Figure 17). These sources include soil, culture, marine (sediment, water, sponge, coral, and algae), plants, fresh water, and other sources (Figure 17). In Figure 17, it is clear that the major number of metabolites have been reported from the fungus strains collected from diverse marine sources. Among these metabolites, isocoumarins, diketopiperazines, and polyketides represent the major reported constituents. Additionally, sesquiterpenoids with nitrobenzoyl moiety, a rare class of metabolites, are mainly reported from *Aspergillus* species, including *A. ochraceus*. Therefore, these sesquiterpenoids deserve more attention because of their marked antitumor potential.

Despite the great number of metabolites reported from this fungus, bioactivities were investigated for a limited number of them. The tested bioactivities were antimicrobial, anti-Parkinson’s, antiviral, antioxidant, cytotoxic, neuroprotective, and insecticidal. Some of the reported metabolites possessed potent effectiveness similar to or higher than the positive control, for example, **4** displayed a selectivity towards *V. harveyi* compared to that of chloramphenicol. Compounds **6** and **67** demonstrated more potent antioxidant potential than BHT. In addition, **6** had cytoprotective potential H_2_O_2_-induced oxidative damage in SHSY5Y cells without toxicity, suggesting its possible protective role in neurodegenerative illnesses with oxidative stress. Additionally, **56** featured prominent anti-Parkinson’s effectiveness compared to levodopa, which could be an advantageous lead compound for anti-Parkinson’s therapeutic drugs. Moreover, some metabolites were reported to show strong cytotoxic capacity (e.g., **18**, **76**–**79**, and **91**).

In addition, there is a lack of pharmacological studies that focus on exploring the possible mechanisms of the active metabolites. In addition, the untested metabolites should be further explored for their possible bioactivities.

Using the OSMAC technique on the sponge-derived *A. ochraceus* yielded waspergillamide B (**39**), a new metabolite with uncommon structural features. In addition, modification of culture media—for example by adding NaBr or NaI—resulted in the production of new metabolites, e.g., (*R*)-(–)-5-bromomellein (**5**) and ochramides A–D (**31**–**34**), respectively, while mutation of the *A. ochraceus* strain by UV irradiation produced new metabolites (e.g., **155** and **163**). In the same line, the co-cultivation with bacterial strains, such as *S. lividans*, increased the yield (e.g., **97** and **98**); however, *B. subtilis* yielded new derivatives (e.g., ochraspergillic acids A (**123**) and B (**124**)). This suggests further studies on utilizing these techniques could be performed.

Furthermore, the few reports on the biosynthesis of these metabolites promote further study for exploring the biosynthetic pathways of other metabolites and the associated genes that support the potential of *A. ochraceus* for novel metabolite production using metabolic engineering. Only one study reported the synthesis of NPs using this fungus; further research on synthesizing various types of NPs and its bio-evaluation represents an interesting area for more expected valuable impacts. Additionally, the enzyme production capacity of this fungus has been approved in various studies, suggesting its potential for industrial and biotechnological applications. Thus, *A. ochraceus* could represent an eco-friendly tool for exchanging hazardous waste into valuable products. Despite the considerable published studies, this fungus still needs more in-depth research from mycologists, biologists, and chemists to shed light on the unexplored potential of this fungus and its metabolites.

## Figures and Tables

**Table 1:** Secondary metabolites reported from *Aspergillus ochraceus* (chemical class, molecular weight and formulae, fungal source, host, and place).

Compound Name/Chemical Class	Mol. Wt.	Mol. Formula	Host (Part, Family)/Location	Ref.
Isocoumarin derivatives				
3*R*-Mellein (**1**)	178	C_10_H_10_O_3_	Wet maize meal	[77,78]
	-	-	Sea of Japan sediment	[20]
	-	-	*Chondria crassicualis* (Red alga, Rhodomelaceae), Yokji Island, Kyeongnam, Korea	[72]
	-	-	*Dichotella gemmacea* (Coral, Ellisellidae), South Sea, China	[79]
(3*R*,4*S*)-4-Hydroxymellein (**2**)	194	C_10_H_10_O_4_	Cultured	[16,78]
	-	-	Rhizospheric soil of *Bruguiera gymnorrhiza* (Marine mangrove plant, Rhizophoraceae), Hainan Island, China	[71]
	-	-	Pacific Ocean, China	[21]
(3*R*,4*R*)-4-Hydroxymellein (**3**)	194	C_10_H_10_O_4_	Rhizospheric soil of *Bruguiera gymnorrhiza* (Marine mangrove plant, Rhizophoraceae), Hainan Island, China	[71]
(*R*)-7-Hydroxymellein (**4**)	194	C_10_H_10_O_4_	Rhizospheric soil of *Bruguiera gymnorrhiza* (Marine mangrove plant, Rhizophoraceae), Hainan Island, China	[71]
(*R*)-(–)-5-Bromomellein (**5**)	255	C_10_H_9_BrO_3_	*Chondria crassicualis* (Red alga, Rhodomelaceae), Yokji Island, Kyeongnam, Korea	[72]
(±)-4,7-Dihydroxymellein (**6**)	210	C_10_H_10_O_5_	Pacific Ocean, China	[21]
(3*R*,4*S*)-3,4-Dihydro-4,5,8-trihydroxy-3-methyl-1*H*-2-benzopyran-1-one (**7**)	210	C_10_H_10_O_5_	Deep-sea water, Northeastern Pacific, China	[80]
6-Methoxy-8-hydroxyisocoumarin-3-carboxylic acid (**8**)	236	C_11_H_8_O_6_	Cultured	[73]
(±)-Botryoisocoumarin A (**9**)	208	C_11_H_12_O_4_	Rhizospheric soil of *Bruguiera gymnorrhiza* (Marine mangrove plant, Rhizophoraceae), Hainan Island, China	[71]
Diaporthin (**10**)	250	C_13_H_14_O_5_	Cultured	[81]
Orthosporin (**11**)	236	C_12_H_12_O_5_	Cultured	[81]
Ochratoxin α (**12**)	256	C_11_H_9_ClO_5_	Cultured	[76]
Ochratoxin β (**13**)	222	C_11_H_10_O_5_	Cultured	[76]
Ochratoxin A (**14**)	403	C_20_H_18_ClNO_6_	Wet maize meal	[74,75,77]
	-	-	*Agelas oroides* (Sponge Agelasidae), Sığaçık-Izmir, Turkey	[17]
4-*R*-Hydroxyochratoxin A (**15**)	419	C_20_H_18_ClNO_7_	Cultured	[76]
4-*S*-Hydroxyochratoxin A (**16**)	419	C_20_H_18_ClNO_7_	Cultured	[76]
10-Hydroxyochratoxin A (**17**)	419	C_20_H_18_ClNO_7_	Cultured	[76]
Ochratoxin B (**18**)	369	C_20_H_19_NO_6_	Wet maize meal	[75,77]
	-	-	*Agelas oroides* (Sponge Agelasidae), Sığaçık-Izmir, Turkey	[17]
Bromo-ochratoxin B (**19**)	447	C_20_H_18_BrNO_6_	Cultured	[82]
4-*R*-Hydroxyochratoxin B (**20**)	385	C_20_H_19_NO_7_	Cultured	[76]
Ochratoxin C (**21**)	431	C_22_H_22_ClNO_6_	Wet maize meal	[75,77]
HO-Proline-ochratoxin (**22**)	369	C_16_H_16_ClNO_7_	Cultured	[83]
Serine-ochratoxin (**23**)	343	C_14_H_14_ClNO_7_	Cultured	[83]
Lysine-ochratoxin (**24**)	384	C_17_H_21_ClN_2_O_6_	Cultured	[83]
Pyrazine derivatives				
Flavacol (**25**)	208	C_12_H_20_N_2_O	Moldy rice	[84,85]
	-	-	Laboratory infected soil	[86]
	-	-	*Dichotella gemmacea* (Coral, Ellisellidae), South China Sea, Lingao, Hainan, China	[22]
6-(2-Hydroxy-2-methylpropyl)-3-isobutylpyrazin-2-ol (**26**)	224	C_12_H_20_N_2_O_2_	Moldy rice	[84]
Neoaspergillc acid (**27**)	224	C_12_H_20_N_2_O_2_	Moldy rice	[84,85]
	-	-	*Dichotella gemmacea* (Coral, Ellisellidae), South China Sea, Lingao, Hainan, China	[22]
Neohydroxyaspergillc acid (**28**)	240	C_12_H_20_N_2_O_3_	Moldy rice	[85]
	-	-	*Dichotella gemmacea* (Coral, Ellisellidae), South China Sea, Lingao, Hainan, China	[22]
β-Hydroxyneoaspergillic acid (**29**)	240	C_12_H_20_N_2_O_3_	Moldy rice	[85]
	-	-	*Dichotella gemmacea* (Coral, Ellisellidae), South China Sea, Lingao, Hainan, China	[22]
Deoxy-β-hydroxyneoaspergillic acid (**30**)	224	C_12_H_20_N_2_O_2_	Moldy rice	[84,85]
	-	-	*Dichotella gemmacea* (Coral, Ellisellidae), South China Sea, Lingao, Hainan, China	[22]
Ochramide A (**31**)	224	C_12_H_20_N_2_O_2_	*Dichotella gemmacea* (Coral, Ellisellidae), South China Sea, Lingao, Hainan, China	[22]
Ochramide B (**32**)	238	C_13_H_22_N_2_O_2_	*Dichotella gemmacea* (Coral, Ellisellidae), South China Sea, Lingao, Hainan, China	[22]
Ochramide C (**33**)	254	C_13_H_22_N_2_O_3_	*Dichotella gemmacea* (Coral, Ellisellidae), South China Sea, Lingao, Hainan, China	[22]
Ochramide D (**34**)	238	C_12_H_18_N_2_O_3_	*Dichotella gemmacea* (Coral, Ellisellidae), South China Sea, Lingao, Hainan, China	[22]
3-Isobutyl-6-(1-hydroxy-2-methylpropyl)-2(1*H*)-pyrazinone (**35**)	224	C_12_H_20_N_2_O_2_	*Dichotella gemmacea* (Coral, Ellisellidae), Lingao, Hainan, China	[22]
Ferrineoaspergillin (**36**)	725	C_36_H_57_FeN_6_O_6_	Moldy rice	[84,85]
Ochralate A (**37**)	786	C_39_H_63_AlN_6_O_9_	*Dichotella gemmacea* (Coral, Ellisellidae), South China Sea, Lingao, Hainan, China	[22,86]
Aluminiumneoaspergillin (**38**)	744	C_36_H_57_AlN_6_O_9_	South China Sea *Dichotella gemmacea* (Ellisellidae), Lingao, Hainan, China	[22]
Diketopiperazines				
Waspergillamide B (**39**)	450	C_20_H_26_N_4_O_8_	*Agelas oroides* (Sponge, Agelasidae), Sığaçık-Izmir, Turkey	[17]
Epiamauromine (**40**)	508	C_32_H_36_N_4_O_2_	Cultured	[28]
N-Methylepiamauromine (**41**)	522	C_33_H_38_N_4_O_2_	Cultured	[28]
Cycloechinulin (**42**)	351	C_20_H_21_N_3_O_3_	Cultured	[28,87]
CJ-17,665 = Avrainvillamide (**43**)	445	C_26_H_27_N_3_O_4_	Soil, Venezuela	[88]
Stephacidin A (**44**)	431	C_26_H_29_N_3_O_3_	Light brown clay, Sirsaganj, Uttar Pradesh, India	[89]
	-	-	Laboratory infected soil	[86]
	-	-	Soil, China	[90]
	-	-	*Agelas oroides* (Sponge, Agelasidae), Sığaçık-Izmir, Turkey	[17]
Stephacidin B (**45**)	890	C_52_H_54_N_6_O_8_	Light brown clay, Sirsaganj, Uttar Pradesh, India	[89]
Speramide A (**46**)	447	C_26_H_29_N_3_O_4_	Freshwater, Fuxian Lake, China	[23]
Speramide B (**47**)	465	C_26_H_31_N_3_O_5_	Freshwater, Fuxian Lake, China	[23]
Asperochramide A (**48**)	451	C_26_H_33_N_3_O_4_	Soil, China	[90]
Asperochramide B (**49**)	451	C_26_H_33_N_3_O_4_	Soil, China	[90]
Asperochramide C (**50**)	397	C_21_H_23_N_3_O_5_	Soil, China	[90]
Asperochramide D (**51**)	315	C_16_H_17_N_3_O_4_	Soil, China	[90]
Notoamide A (**52**)	463	C_26_H_29_N_3_O_5_	Deep-sea water, Northeastern Pacific, China	[80]
Notoamide B (**53**)	447	C_26_H_29_N_3_O_4_	*Sargassum kjellmanianum* (Brown alga, Sargassaceae), Dalian coastline, China	[26]
	-	-	Soil, China	[90]
Notoamide C (**54**)	449	C_26_H_31_N_3_O_4_	Soil, China	[90]
Notoamide F (**55**)	461	C_27_H_31_N_3_O_4_	Marine Culture, Pacific Ocean, China	[24]
Notoamide I (**56**)	445	C_26_H_27_N_3_O_4_	Deep-sea water, Northeastern Pacific, China	[80]
Notoamide M (**57**)	465	C_26_H_31_N_3_O_5_	Soil, China	[90]
	-	-	Deep-sea water, Northeastern Pacific, China	[80]
Notoamide X (**58**)	461	C_26_H_27_N_3_O_5_	Deep-sea water, Northeastern Pacific, China	[80]
Sclerotiamide (**59**)	463	C_26_H_29_N_3_O_5_	Deep-sea water, Northeastern Pacific, China	[80]
Taichunamide D (**60**)	509	C_27_H_31_N_3_O_5_S	Soil, China	[90]
Versicolamide B (**61**)	447	C_26_H_29_N_3_O_4_	Marine Culture, Pacific Ocean, China	[24]
Brevianamide F (**62**)	283	C_16_H_17_N_3_O_2_	Soil, China	[90]
Benzodiazepines				
(11a*S*)-2,3-Dihydro-7-methoxy-1*H*-pyrrolo [2,1-*c*][1,4] benzodiazepine-5,11(10*H*,11a*H*)-dione (**63**)	246	C_13_H_14_N_2_O_3_	*Sargassum kjellmanianum* (Brown alga, Sargassaceae), Dalian coastline, China	[26]
Circumdatin A (**64**)	363	C_20_H_17_N_3_O_4_	Sterilized milo sorghum seeds buried in the soil for 1–4 months, Sevilleta National Wildlife Refuge, Socorro County, New Mexico, USA	[91]
	-	-	*Chondria crassicualis* (Red alga, Rhodomelaceae), Yokji Island, Kyeongnam, Korea	[72]
Circumdatin B (**65**)	393	C_21_H_19_N_3_O_5_	Sterilized milo sorghum seeds buried in the soil for 1–4 months, Sevilleta National Wildlife Refuge, Socorro County, New Mexico, USA	[91]
	-	-	*Agelas oroides* (Sponge Agelasidae), Sığaçık-Izmir, Turkey	[17]
Circumdatin C (**66**)	307	C_17_H_13_N_3_O_3_	Sterilized milo sorghum seeds buried in the soil for 1–4 months, Sevilleta National Wildlife Refuge, Socorro County, New Mexico, USA	[91]
	-	-	Sea of Japan sediment	[20]
	-	-	*Sargassum kjellmanianum* (Brown alga, Sargassaceae), Dalian coastline, China	[26]
2-Hydroxycircumdatin C (**67**)	323	C_17_H_13_N_3_O_4_	*Sargassum kjellmanianum* (Brown alga, Sargassaceae), Dalian coastline, China	[26]
	-	-	*Dichotella gemmacea* (Coral, Ellisellidae), Lingao, Hainan, China	[31]
Circumdatin D (**68**)	393	C_21_H_19_N_3_O_5_	Sterilized milo sorghum seeds buried in the soil for 1–4 months, Sevilleta National Wildlife Refuge, Socorro County, New Mexico, USA	[92]
	-	-	Laboratory infected soil	[86]
	-	-	*Sargassum kjellmanianum* (Brown alga, Sargassaceae), Dalian coastline, China	[26]
Circumdatin E (**69**)	363	C_20_H_17_N_3_O_4_	Sterilized milo sorghum seeds buried in the soil for 1–4 months, Sevilleta National Wildlife Refuge, Socorro County, New Mexico, USA	[92]
	-	-	Laboratory infected soil	[86]
	-	-	*Agelas oroides* (Sponge Agelasidae), Sığaçık-Izmir, Turkey	[17]
Circumdatin F (**70**)	291	C_17_H_13_N_3_O_2_	Sterilized milo sorghum seeds buried in the soil for 1–4 months, Sevilleta National Wildlife Refuge, Socorro County, New Mexico, USA	[92]
	-	-	Sea of Japan sediment	[20]
	-	-	*Sargassum kjellmanianum* (Brown alga, Sargassaceae), Dalian coastline, China	[26]
	-	-	*Agelas oroides* (Sponge, Agelasidae), Sığaçık-Izmir, Turkey	[17]
Circumdatin G (**71**)	307	C_17_H_13_N_3_O_3_	Sea of Japan sediment	[20]
	-	-	*Agelas oroides* (Sponge, Agelasidae), Sığaçık-Izmir, Turkey	[17]
Circumdatin H (**72**)	347	C_20_H_17_N_3_O_3_	Laboratory infected soil	[86]
	-	-	*Agelas oroides* (Sponge, Agelasidae), Sığaçık-Izmir, Turkey	[17]
Circumdatin I (**73**)	323	C_17_H_13_N_3_O_4_	Cultured	[34]
Circumdatin L (**74**)	307	C_17_H_13_N_3_O_3_	*Agelas oroides* (Sponge, Agelasidae), Sığaçık-Izmir, Turkey	[17]
Circumdatin N (**75**)	307	C_17_H_13_N_3_O_3_	Marine Culture, Pacific Ocean, China	[24]
Ochrazepine A (**76**)	507	C_26_H_25_N_3_O_8_	*Dichotella gemmacea* (Coral, Ellisellidae), Lingao, Hainan, China	[31]
Ochrazepine B (**77**)	507	C_26_H_25_N_3_O_8_	*Dichotella gemmacea* (Coral, Ellisellidae), Lingao, Hainan, China	[31]
Ochrazepine C (**78**)	507	C_26_H_25_N_3_O_8_	*Dichotella gemmacea* (Coral, Ellisellidae), Lingao, Hainan, China	[31]
Ochrazepine D (**79**)	507	C_26_H_25_N_3_O_8_	*Dichotella gemmacea* (Coral, Ellisellidae), Lingao, Hainan, China	[31]
Indole and other alkaloids				
Ochrindole A (**80**)	452	C_29_H_28_N_2_O_3_	Cultured	[29]
Ochrindole B (**81**)	468	C_29_H_28_N_2_O_4_	Cultured	[29]
Ochrindole C (**82**)	438	C_28_H_26_N_2_O_3_	Cultured	[29]
Ochrindole D (**83**)	422	C_27_H_22_N_2_O_3_	Cultured	[29]
Perlolyrine (**84**)	264	C_16_H_12_N_2_O_2_	Marine Culture, Pacific Ocean, China	[24]
Ochracesol A (**85**)	205	C_11_H_11_NO_3_	Marine Culture, Pacific Ocean, China	[24]
L-657,398 (**86**)	317	C_21_H_35_NO	Cultured	[93]
Cycloanthranilylproline (**87**)	230	C_13_H_14_N_2_O_2_	*Agelas oroides* (Sponge, Agelasidae), Sığaçık-Izmir, Turkey	[17]
Peptides				
Aspochracin (**88**)	432	C_23_H_36_N_4_O_4_	Cultured	[94,95,96]
Violaceotide A (**89**)	476	C_24_H_36_N_4_O_6_	*Agelas oroides* (Sponge, Agelasidae), Sığaçık-Izmir, Turkey	[17]
Sesquiterpenoids				
6β,9α-Dihydroxy-14-*p*-nitrobenzoylcinnamolide (**90**)	431	C_22_H_25_NO_8_	*Coelarthrum* sp. (Green alga, Rhodymeniaceae), South China Sea, China	[32,97]
Insulicolide A (**91**)	431	C_22_H_25_NO_8_	*Coelarthrum* sp. (Green alga, Rhodymeniaceae), South China Sea, China	[32]
14-O-Acetylinsulicolide A (**92**)	475	C_23_H_25_NO_10_	*Coelarthrum* sp. (Green alga, Rhodymeniaceae), South China Sea, China	[97]
9-Deoxyinsulicolide A (**93**)	415	C_22_H_25_NO_7_	*Coelarthrum* sp. (Green alga, Rhodymeniaceae), South China Sea, China	[97]
Insulicolide B (**94**)	415	C_22_H_25_NO_7_	*Coelarthrum* sp. (Green alga, Rhodymeniaceae), South China Sea, China	[97]
Insulicolide C (**95**)	459	C_23_H_25_NO_9_	*Coelarthrum* sp. (Green alga, Rhodymeniaceae), South China Sea, China	[97]
6β,14-Dihydroxy-7α-methoxyconfertifolin (**96**)	296	C_16_H_24_O_5_	*Coelarthrum* sp. (Green alga, Rhodymeniaceae), South China Sea, China	[97]
Polyketides				
Penicillic acid (**97**)	170	C_8_H_10_O_4_	Cultured	[30,98]
	-	-	Rhizospheric soil of *Bruguiera gymnorrhiza* (Mangrove plant Rhizophoraceae), Hainan Island, China	[71]
	-	-	*Agelas oroides* (Sponge Agelasidae), Sığaçık-Izmir, Turkey	[17]
5(6)-Dihydropenicillic acid (**98**)	172	C_8_H_12_O_4_	Cultured	[98]
	-	-	Rhizospheric soil of *Bruguiera gymnorrhiza* (Mangrove plant Rhizophoraceae), Hainan Island, China	[71]
	-	-	*Agelas oroides* (Sponge Agelasidae), Sığaçık-Izmir, Turkey	[17]
Asperlactone (**99**)	184	C_9_H_12_O_4_	Environmental contamination sample, apple-packing house, Lleida, Spain	[99]
Chlorohydroasperlactone A (**100**)	220	C_9_H_13_ClO_4_	Rhizospheric soil of *Bruguiera gymnorrhiza* (Mangrove plant Rhizophoraceae), Hainan Island, China	[71]
Chlorohydroasperlactone B (**101**)	220	C_9_H_13_ClO_4_	Rhizospheric soil of *Bruguiera gymnorrhiza* (Mangrove plant Rhizophoraceae), Hainan Island, China	[71]
	-	-	*Dichotella gemmacea* (Coral, Ellisellidae), South Sea, China	[79]
Aspilactonol B (**102**)	202	C_9_H_14_O_5_	Deep-sea water, Northeastern Pacific, China	[80]
Aspilactonol E (**103**)	186	C_9_H_14_O_4_	Deep-sea water, Northeastern Pacific, China	[80]
Asperochrin A (**104**)	202	C_9_H_14_O_5_	Rhizospheric soil of *Bruguiera gymnorrhiza* (Mangrove plant, Rhizophoraceae), Hainan Island, China	[71]
Asperochrin B (**105**)	184	C_9_H_12_O_4_	Rhizospheric soil of *Bruguiera gymnorrhiza* (Mangrove plant, Rhizophoraceae), Hainan Island, China	[71]
Asperochrin C (**106**)	184	C_9_H_12_O_4_	Rhizospheric soil of *Bruguiera gymnorrhiza* (Mangrove plant Rhizophoraceae), Hainan Island, China	[71]
Asperochratide A (**107**)	216	C_10_H_16_O_5_	Deep-sea water, Northeastern Pacific, China	[80]
Asperochratide B (**108**)	202	C_9_H_14_O_5_	Deep-sea water, Northeastern Pacific, China	[80]
Asperochratide C (**109**)	216	C_10_H_16_O_5_	Deep-sea water, Northeastern Pacific, China	[80]
Asperochratide D (**110**)	202	C_9_H_14_O_5_	Deep-sea water, Northeastern Pacific, China	[80]
Asperochratide E(**111**)	202	C_9_H_14_O_5_	Deep-sea water, Northeastern Pacific, China	[80]
Asperochratide F (**112**)	202	C_9_H_14_O_5_	Deep-sea water, Northeastern Pacific, China	[80]
Asperochratide G (**113**)	202	C_9_H_14_O_5_	Deep-sea water, Northeastern Pacific, China	[80]
Asperochratide H (**114**)	188	C_9_H_16_O_4_	Deep-sea water, Northeastern Pacific, China	[80]
Asperochratide I (**115**)	202	C_9_H_14_O_5_	Deep-sea water, Northeastern Pacific, China	[80]
Asperochratide J (**116**)	216	C_10_H_16_O_5_	Deep-sea water, Northeastern Pacific, China	[80]
Aspyrone (**117**)	184	C_9_H_12_O_4_	Cultured	[33,100,101,102]
	-	-	Environmental contamination sample, apple-packing house, Lleida, Spain	[99]
	-	-	*Dichotella gemmacea* (Coral, Ellisellidae), Lingao, Hainan, China	[31]
Chlorohydroaspyrone A (**118**)	220	C_9_H_13_ClO_4_	Rhizospheric soil of *Bruguiera gymnorrhiza* (Mangrove plant Rhizophoraceae), Hainan Island, China	[71]
Chlorohydroaspyrone B (**119**)	220	C_9_H_13_ClO_4_	Rhizospheric soil of *Bruguiera gymnorrhiza* (Mangrove plant Rhizophoraceae), Hainan Island, China	[71]
	-	-	*Dichotella gemmacea* (Coral, Ellisellidae), South Sea, China	[79]
Aspyronol (**120**)	216	C_10_H_16_O_5_	Rhizospheric soil of *Bruguiera gymnorrhiza* (Mangrove plant Rhizophoraceae), Hainan Island, China	[71]
Dihydroaspyrone (**121**)	186	C_9_H_14_O_4_	Cultured	[33]
	-	-	Rhizospheric soil of *Bruguiera gymnorrhiza* (Mangrove plant Rhizophoraceae), Hainan Island, China	[71]
	-	-	*Dichotella gemmacea* (Coral, Ellisellidae), South Sea, China	[79]
	-	-	*Agelas oroides* (Sponge, Agelasidae), Sığaçık-Izmir, Turkey	[17]
8,9-Dihydroxy-8,9-deoxyaspyrone (**122**)	202	C_9_H_14_O_5_	Deep-sea water, Northeastern Pacific, China	[80]
Ochraspergillic acid A (**123**)	307	C_15_H_17_NO_6_	*Agelas oroides* (Sponge, Agelasidae), Sığaçık-Izmir, Turkey	[17]
Ochraspergillic acid B (**124**)	307	C_15_H_17_NO_6_	*Agelas oroides* (Sponge Agelasidae), Sığaçık-Izmir, Turkey	[17]
Aspinolide A (**125**)	184	C_10_H_16_O_3_	Cultured	[33]
Aspinolide B (**126**)	284	C_14_H_20_O_6_	Cultured	[33]
Aspinolide C (**127**)	282	C_14_H_18_O_6_	Cultured	[33]
Aspinonene (**128**)	188	C_9_H_16_O_4_	Cultured	[33,100]
*Iso*-aspinonene (**129**)	188	C_9_H_16_O_4_	Cultured	[33]
Quinone Derivatives				
Emodin (**130**)	270	C_15_H_10_O_5_	Moldy rice	[103]
			*Solanum tuberosum* (Chinese potato, Solanaceae)	[104]
Xanthomegnin (**131**)	574	C_30_H_22_O_12_	Cultured	[105,106]
	-	-	*Agelas oroides* (Sponge, Agelasidae), Sığaçık-Izmir, Turkey	[17]
Viomellein (**132**)	560	C_30_H_24_O_11_	Cultured	[105,106]
	-	-	*Agelas oroides* (Sponge, Agelasidae), Sığaçık-Izmir, Turkey	[17]
Xanthine Derivatives				
Vioxanthin (**133**)	546	C_30_H_26_O_10_	Green coffee beans	[107]
*R*-(+)-*Semi*-vioxanthin (**134**)	274	C_15_H_14_O_5_	*Dichotella gemmacea* (Coral, Ellisellidae), South Sea, China	[79]
Secalonic acid (**135**)	638	C_32_H_30_O_14_	Moldy rice	[103]
Flavonoids				
Lucenin-2 (**136**)	610	C_27_H_30_O_16_	Marine sponge, Kanyakumari, peninsular land, southeast coast of Tamilnadu state, India	[108]
Sterols				
7-Nor-ergosterolide (**137**)	414	C_27_H_42_O_3_	*Sargassum kjellmanianum* (Brown alga, Sargassaceae), Dalian coastline, China	[27]
3β,11α-Dihydroxyergosta-8,24(28)-dien-7-one (**138**)	428	C_28_H_44_O_3_	*Sargassum kjellmanianum* (Brown alga, Sargassaceae), Dalian coastline, China	[27]
3β-Hydroxyergosta-8,24(28)-dien-7-one (**139**)	412	C_28_H_44_O_2_	*Sargassum kjellmanianum* (Brown alga, Sargassaceae), Dalian coastline, China	[27]
(22*E*,24*R*)-3β,5α,9α-Trihydroxyergosta-7,22-dien-6-one (**140**)	444	C_28_H_44_O_4_	*Sargassum kjellmanianum* (Brown alga, Sargassaceae), Dalian coastline, China (Sargassaceae), Dalian coastline, China	[27]
(22*E*,24*R*)-3β,5α -Dihydroxyergosta-7,22-dien-6-one (**141**)	428	C_28_H_44_O_3_	*Sargassum kjellmanianum* (Brown alga, Sargassaceae), Dalian coastline, China	[27]
Ergosterol (**142**)	396	C_28_H_44_O	*Sargassum kjellmanianum* (Brown alga, Sargassaceae), Dalian coastline, China	[27]
(22*E*,24*R*)-Ergosta-4,6,8(14),22-tetraen-3-one (**143**)	392	C_28_H_40_O	*Sargassum kjellmanianum* (Brown alga, Sargassaceae), Dalian coastline, China	[27]
(22*E*,24*R*)-Ergosta-7,22-diene-3β,5α,6β-triol (**144**)	430	C_28_H_46_O_3_	*Sargassum kjellmanianum* (Brown alga, Sargassaceae), Dalian coastline, China	[27]
(22*E*,24*R*)-Ergosta-7,22-diene-6β-methoxy-3β,5*α*-diol (**145**)	444	C_29_H_48_O_3_	*Sargassum kjellmanianum* (Brown alga, Sargassaceae), Dalian coastline, China	[27]
(22*E*,24*R*)-ergosta-7,22-diene-3β,6β-diol (**146**)	414	C_28_H_46_O_2_	*Sargassum kjellmanianum* (Brown alga, Sargassaceae), Dalian coastline, China	[27]
(22*E*,24*R*)-Ergosta-5*α*,6*α*-epoxide-8,22-diene-3β,7*α*-diol (**147**)	428	C_28_H_44_O_3_	*Sargassum kjellmanianum* (Brown alga, Sargassaceae), Dalian coastline, China	[27]
(22*E*,24*R*)-5*α*,8*α*-Epidioxyergosta-6,22-dien-3β-ol (**148**)	428	C_28_H_44_O_3_	*Sargassum kjellmanianum* (Brown alga, Sargassaceae), Dalian coastline, China	[27]
(22*E*,24*R*)-Ergosta-7,22-diene-3β,5α,6β,9α-tetraol (**149**)	446	C_28_H_46_O_4_	Deep-sea water, Northeastern Pacific, China	[80]
Ochrasterone (**150**)	456	C_30_H_48_O_3_	Pacific Ocean, China	[21]
Gymnasterone D (**151**)	406	C_28_H_38_O_2_	Marine Culture, Pacific Ocean, China	[24]
Isocyathisterol (**152**)	410	C_28_H_42_O_2_	Marine Culture, Pacific Ocean, China	[24]
Herbarulide (**153**)	424	C_28_H_40_O_3_	Marine Culture, Pacific Ocean, China	[24]
Demethylincisterol A2 (**154**)	332	C_21_H_32_O_3_	Marine Culture, Pacific Ocean, China	[24]
Other Metabolites				
4-(3-Methyl-2- butenyl) oxy-1-phenyl acetic acid (**155**)	220	C_13_H_16_O_3_	Cultured	[109]
Clavatol (**156**)	180	C_10_H_12_O_3_	*Chondria crassicualis* (Korean name: Seosil) (Red alga, Rhodomelaceae), Yokji Island, Kyeongnam, Korea	[72]
2,2-[bis-4-(2,3-Dihydroxypropoxy) phenyl]propane (**157**)	376	C_21_H_28_O_6_	Deep-sea water of the Northeastern Pacific, China	[80]
6-Ethyloct-3-yl-2- ethylhexyl ester (**158**)	418	C_26_H_42_O_4_	Marine sponge, Kanyakumari, peninsular land, southeast coast of Tamilnadu state, India	[108]
Di-(2-Ethylhexyl) phthalate (**159**)	390	C_24_H_38_O_4_	Marine Culture, Pacific Ocean, China	[24]
Campholene aldehyde (**160**)	152	C_10_H_16_O	Marine sponge, Kanyakumari, peninsular, southeast coast of Tamilnadu state, India	[108]
Dientriol (**161**)	172	C_9_H_16_O_3_	Cultured	[33]
Triendiol (**162**)	154	C_9_H_14_O_2_	Cultured	[33]
2,10-Dimethyl 4-hydroxy-6-oxo-4-undecen-7-yne (**163**)	208	C_13_H_20_O_2_	Cultured	[109]
Eythrol (**164**)	122	C_4_H_10_O_4_	Deep-sea water, Northeastern Pacific, China	[80]
Galactomannan (**165**)	828	C_30_H_52_O_26_	*Dichotella gemmacea* (Coral, Ellisellidae), South Sea, China	[35]

**Table 2:** Biological activity of reported metabolites from *Aspergillus ochraceus*.

Compound Name	Biological Activity	Assay, Organism or Cell Line	Biological Results		Ref.
			Compound	Positive Control	
3*R*-Mellein (**1**)	Anti HCV protease	SPA	35.0 µM (IC_50_)	-	[20]
	Antioxidant	Spectrophotometry/DPPH	25.0 µM (IC_50_)	l-ascorbic acid 20.0 μM (IC_50_)	[72]
(3*R*,4*S*)-4-Hydroxymellein (**2**)	Antioxidant	Spectrophotometry/DPPH	129.36 µM (IC_50_)	BHT 91.35 µM (IC_50_)	[21]
		Spectrophotometry/ABTS	140.23 µM (IC_50_)	Trolox 101.23 µM (IC_50_)	[21]
		Spectrophotometry/FRAP	11.96 µM (IC_50_)	Trolox 1.80 µM (IC_50_)	[21]
(*R*)-7-Hydroxymellein (**4**)	Antibacterial	Well diffusion/*A. hydrophilia*	64.0 μg/mL (MIC)	Chloramphenicol 4.0 μg/mL (MIC)	[71]
		Well diffusion/*V. anguillarum*	32.0 μg/mL (MIC)	Chloramphenicol 1.0 μg/mL (MIC)	[71]
		Well diffusion/*V. harveyi*	8.0 μg/mL (MIC)	Chloramphenicol 8.0 μg/mL (MIC)	[71]
(*R*)-(–)-5-Bromomellein (**5**)	Antioxidant	Spectrophotometry/DPPH	24.0 µM (IC_50_)	l-ascorbic acid 20.0 μM (IC_50_)	[72]
(*±*)-4,7-dihydroxymellein (**6**)	Antioxidant	Spectrophotometry/DPPH	62.90 µM (IC_50_)	BHT 91.35 µM (IC_50_)	[21]
		Spectrophotometry/ABTS	70.92 µM (IC_50_)	Trolox 101.23 µM (IC_50_)	[21]
		Spectrophotometry/FRAP	2.53 µM (IC_50_)	Trolox 1.80 µM (IC_50_)	[21]
Ochratoxin B (**18**)	Cytotoxicity	MTT/A2780	3.0 µM (IC_50_)	Cisplatin 2.2 µM (IC_50_)	[17]
Flavacol (**25**)	Inhibition of mitochondrial NADH oxidase	SMP/NADH oxidase	34.6 µM (IC_50_)	-	[86]
CJ-17,665 = Avrainvillamide (**43**)	Antibacterial	Microdilution/*S. aureus*	12.5 μg/mL (MIC)	Vancomycin 1.56 μg/mL (MIC)	[88]
		Microdilution/*S. pyogenes*	12.5 μg/mL (MIC)	Vancomycin 0.39 μg/mL (MIC)	[88]
		Microdilution/*E. pyogenes*	25.0 μg/mL (MIC)	Vancomycin 12.5 μg/mL (MIC)	[88]
	Cytotoxicity	MTT/HeLa	1.1 μg/mL (IC_90_)	-	[88]
Stephacidin A (**44**)	Cytotoxicity	MTT/PC-3	2.1 µM (IC_50_)	-	[89]
		MTT/LNCaP	1.0 µM (IC_50_)	-	[89]
		MTT/A2780	4.0 µM (IC_50_)	-	[89]
		MTT/A2780/DDP	6.8 µM (IC_50_)	-	[89]
		MTT/A2780/Tax	3.6 µM (IC_50_)	-	[89]
		MTT/HCT-116	2.1 µM (IC_50_)	-	[89]
		MTT/HCT116/mdr+	6.7 µM (IC_50_)	-	[89]
		MTT/HCT116/topo	13.1 µM (IC_50_)	-	[89]
		MTT/MCF-7	4.2 µM (IC_50_)	-	[89]
		MTT/SKBR3	2.15 µM (IC_50_)	-	[89]
		MTT/LX-1	4.22 µM (IC_50_)	-	[89]
	Inhibition of mitochondrial NADH Oxidase	SMP/NADH oxidase	13.0 µM (IC_50_)	-	[86]
	Anti-Parkinson’s disease	MPP^+^-induced SH-SY5Y cells	2.45 µM (EC_50_)	Levodopa 2.06 µM (EC_50_)	[24]
Stephacidin B (**45**)	Cytotoxicity	MTT/PC-3	0.37 µM (IC_50_)	-	[89]
		MTT/LNCaP	0.06 µM (IC_50_)	-	[89]
		MTT/A2780	0.33 µM (IC_50_)	-	[89]
		MTT/A2780/DDP	0.43 µM (IC_50_)	-	[89]
		MTT/A2780/Tax	0.26 µM (IC_50_)	-	[89]
		MTT/HCT-116	0.46 µM (IC_50_)	-	[89]
		MTT/HCT116/mdr+	0.46 µM (IC_50_)	-	[89]
		MTT/HCT116/topo	0.42 µM (IC_50_)	-	[89]
		MTT/MCF-7	0.27 µM (IC_50_)	-	[89]
		MTT/SKBR3	0.32 µM (IC_50_)	-	[89]
		MTT/LX-1	0.38 µM (IC_50_)	-	[89]
Speramide A (**46**)	Antibacterial	2-Fold dilution/ *P. aeruginosa*	0.8 µM (MIC)	-	[23]
Notoamide B (**53**)	Anti-Parkinson’s disease	MPP^+^--induced SH-SY5Y cells	5.31 µM (EC_50_)	Levodopa 2.06 µM (EC_50_)	[24]
Notoamide C (**54**)	Anti-Parkinson’s disease	MPP^+^--induced SH-SY5Y cells	7.39 µM (EC_50_)	Levodopa 2.06 µM (EC_50_)	[24]
Notoamide F (**55**)	Anti-Parkinson’s disease	MPP^+^--induced SH-SY5Y cells	2.98 µM (EC_50_)	Levodopa 2.06 µM (EC_50_)	[24]
Notoamide I (**56**)	Anti-Parkinson’s disease	MPP^+^--induced SH-SY5Y cells	2.30 µM (EC_50_)	Levodopa 2.06 µM (EC_50_)	[24]
Versicolamide B (**61**)	Anti-Parkinson’s disease	MPP^+^--induced SH-SY5Y cells	6.01 µM (EC_50_)	Levodopa 2.06 µM (EC_50_)	[24]
Circumdatin A (**64**)	Antioxidant	Spectrophotometry/DPPH	32.0 µM (IC_50_)	l-ascorbic acid 20.0 μM (IC_50_)	[72]
2-Hydroxycircumdatin C (**67**)	Antioxidant	Spectrophotometry/DPPH	9.9 µM (IC_50_)	BHT 88.2 µM (IC_50_)	[26]
	Cytotoxicity	CTG/U251	8.95 µM (IC_50_)	Adramycin 0.19 µM (IC_50_)	[31]
Circumdatin E (**69**)	Inhibition of Mitochondrial NADH Oxidase	SMP/NADH oxidase	2.5 µM (IC_50_)	-	[86]
Circumdatin F (**70**)	Anti-Parkinson’s disease	MPP^+^--induced SH-SY5Y cells	5.44 µM (EC_50_)	Levodopa 2.06 µM (EC_50_)	[24]
Circumdatin G (**71**)	Anti-Parkinson’s disease	MPP^+^--induced SH-SY5Y cells	7.39 µM (EC_50_)	Levodopa 2.06 µM (EC_50_)	[24]
Circumdatin H (**72**)	Inhibition of Mitochondrial NADH Oxidase	SMP/NADH oxidase	1.5 µM (IC_50_)	-	[86]
Circumdatin N (**75**)	Anti-Parkinson’s disease	MPP^+^-induced SH-SY5Y cells	10.77 µM (EC_50_)	Levodopa 2.06 µM (EC_50_)	[24]
Ochrazepine A (**76**)	Cytotoxicity	CTG/MV-4-11	3.94 µM (IC_50_)	Adramycin 0.16 µM (IC_50_)	[31]
		CTG/K562	6.05 µM (IC_50_)	Adramycin 0.02 µM (IC_50_)	[31]
		CTG/A673	3.10 µM (IC_50_)	Adramycin 0.13 µM (IC_50_)	[31]
		CTG/U87	8.67 µM (IC_50_)	Adramycin 0.12 µM (IC_50_)	[31]
		CTG/A549	9.62 µM (IC_50_)	Adramycin 0.10 µM (IC_50_)	[31]
		CTG/N87	6.10 µM (IC_50_)	Adramycin 0.05 µM (IC_50_)	[31]
		CTG/H1299	7.14 µM (IC_50_)	Adramycin 0.49 µM (IC_50_)	[31]
		CTG/HUCCT1	11.32 µM (IC_50_)	Adramycin 0.05 µM (IC_50_)	[31]
		CTG/B16F10	11.22 µM (IC_50_)	Adramycin 0.02 µM (IC_50_)	[31]
		CTG/Karpass299	5.89 µM (IC_50_)	Adramycin 0.39 µM (IC_50_)	[31]
		CTG/HEK-293F	12.91 µM (IC_50_)	Adramycin 0.05 µM (IC_50_)	[31]
		CTG/L02	27.42 µM (IC_50_)	Adramycin 0.10 µM (IC_50_)	[31]
Ochrazepine B (**77**)	Cytotoxicity	CTG/U251	9.91 µM (IC_50_)	Adramycin 0.19 µM (IC_50_)	[31]
		CTG/HEK-293F	73.96 µM (IC_50_)	Adramycin 0.05 µM (IC_50_)	[31]
Ochrazepine C (**78**)	Cytotoxicity	CTG/A673	8.24 µM (IC_50_)	Adramycin 0.13 µM (IC_50_)	[31]
		CTG/U87	9.04 µM (IC_50_)	Adramycin 0.12 µM (IC_50_)	[31]
		CTG/HepB3	10.28 µM (IC_50_)	Adramycin 17.58 µM (IC_50_)	[31]
		CTG/HEK-293F	73.03 µM (IC_50_)	Adramycin 0.05 µM (IC_50_)	[31]
Ochrazepine D (**79**)	Cytotoxicity	CTG/U251	8.26 µM (IC_50_)	Adramycin 0.19 µM (IC_50_)	[31]
		CTG/HEK-293F	54.58 µM (IC_50_)	Adramycin 0.05 µM (IC_50_)	[31]
Perlolyrine (**84**)	Anti-Parkinson’s disease	MPP^+^--induced SH-SY5Y cells	9.97 µM (EC_50_)	Levodopa 2.06 µM (EC_50_)	[24]
Ochracesol A (**85**)	Anti-Parkinson’s disease	MPP^+^--induced SH-SY5Y cells	17.84 µM (EC_50_)	Levodopa 2.06 µM (EC_50_)	[24]
6β,9α-Dihydroxy-14-*p*-nitrobenzoylcinnamolide (**90**)	Cytotoxicity	CCK-8/H1975	2.08 µM (IC_50_)	Trichostatin A 0.09 µM (IC_50_)	[32]
		CCK-8/U937	1.95 µM (IC_50_)	Trichostatin A 0.05 µM (IC_50_)	[32]
		CCK-8/K562	4.33 µM (IC_50_)	Trichostatin A 0.16 µM (IC_50_)	[32]
		CCK-8/BGC823	2.32 µM (IC_50_)	Trichostatin A 0.08 µM (IC_50_)	[32]
		CCK-8/Molt-4	2.39 µM (IC_50_)	Trichostatin A 0.03 µM (IC_50_)	[32]
		CCK-8/MCF-7	4.25 µM (IC_50_)	Trichostatin A 0.06 µM (IC_50_)	[32]
		CCK-8/A549	2.41 µM (IC_50_)	Trichostatin A 0.05 µM (IC_50_)	[32]
		CCK-8/HeLa	6.12 µM (IC_50_)	Trichostatin A 0.10 µM (IC_50_)	[32]
		CCK-8/HL-60	2.44 µM (IC_50_)	Trichostatin A 0.03 µM (IC_50_)	[32]
		CCK-8/Huh-7	3.28 µM (IC_50_)	Trichostatin A 0.09 µM (IC_50_)	[32]
		CCK-8/ACHN	11.0 µM (IC_50_)	Sorafenib 3.4 µM (IC_50_)	[97]
		CCK-8/OS-RC-2	8.2 µM (IC_50_)	Sorafenib 7.0 µM (IC_50_)	[97]
		CCK-8/786-O	4.3 µM (IC_50_)	Sorafenib 4.9 µM (IC_50_)	[97]
	Antiviral	CCK-8/H3N2	17.0 µM (IC_50_)	Oseltamivir A 0.008 µM (IC_50_)	[32]
		CCK-8/EV71	9.4 µM (IC_50_)	Oseltamivir A 0.06 µM (IC_50_)	[32]
Insulicolide A (**91**)	Cytotoxicity	CCK-8/H1975	4.63 µM (IC_50_)	Trichostatin A 0.09 µM (IC_50_)	[32]
		CCK-8/U937	3.97 µM (IC_50_)	Trichostatin A 0.05 µM (IC_50_)	[32]
		CCK-8/K562	4.76 µM (IC_50_)	Trichostatin A 0.16 µM (IC_50_)	[32]
		CCK-8/BGC823	2.78 µM (IC_50_)	Trichostatin A 0.08 µM (IC_50_)	[32]
		CCK-8/Molt-4	2.11 µM (IC_50_)	Trichostatin A 0.03 µM (IC_50_)	[32]
		CCK-8/MCF-7	6.08 µM (IC_50_)	Trichostatin A 0.06 µM (IC_50_)	[32]
		CCK-8/A549	2.86 µM (IC_50_)	Trichostatin A 0.05 µM (IC_50_)	[32]
		CCK-8/HeLa	6.35 µM (IC_50_)	Trichostatin A 0.10 µM (IC_50_)	[32]
		CCK-8/HL-60	2.34 µM (IC_50_)	Trichostatin A 0.03 µM (IC_50_)	[32]
		CCK-8/Huh-7	2.35 µM (IC_50_)	Trichostatin A 0.09 µM (IC_50_)	[32]
		CCK-8/ACHN	1.5 µM (IC_50_)	Sorafenib 3.4 µM (IC_50_)	[97]
		CCK-8/OS-RC-2	1.5 µM (IC_50_)	Sorafenib 7.0 µM (IC_50_)	[97]
		CCK-8/786-O	0.89 µM (IC_50_)	Sorafenib 4.9 µM (IC_50_)	[97]
14-O-Acetylinsulicolide A (**92**)	Cytotoxicity	CCK-8/ACHN	4.1 µM (IC_50_)	Sorafenib 3.4 µM (IC_50_)	[97]
		CCK-8/OS-RC-2	5.3 µM (IC_50_)	Sorafenib 7.0 µM (IC_50_)	[97]
		CCK-8/786-O	2.3 µM (IC_50_)	Sorafenib 4.9 µM (IC_50_)	[97]
9-Deoxyinsulicolide A (**93**)	Cytotoxicity	CCK-8/ACHN	25.0 µM (IC_50_)	Sorafenib 3.4 µM (IC_50_)	[97]
		CCK-8/OS-RC-2	30.0 µM (IC_50_)	Sorafenib 7.0 µM (IC_50_)	[97]
		CCK-8/786-O	20.0 µM (IC_50_)	Sorafenib 4.9 µM (IC_50_)	[97]
Insulicolide B (**94**)	Cytotoxicity	CCK-8/ACHN	30.0 µM (IC_50_)	Sorafenib 3.4 µM (IC_50_)	[97]
		CCK-8/OS-RC-2	23.0 µM (IC_50_)	Sorafenib 7.0 µM (IC_50_)	[97]
		CCK-8/786-O	24.0 µM (IC_50_)	Sorafenib 4.9 µM (IC_50_)	[97]
Insulicolide C (**95**)	Cytotoxicity	CCK-8/ACHN	13.0 µM (IC_50_)	Sorafenib 3.4 µM (IC_50_)	[97]
		CCK-8/OS-RC-2	11.0 µM (IC_50_)	Sorafenib 7.0 µM (IC_50_)	[97]
		CCK-8/786-O	14.0 µM (IC_50_)	Sorafenib 4.9 µM (IC_50_)	[97]
Penicillic acid (**97**)	Antibacterial	Well diffusion/*A. hydrophilia*	1.0 μg/mL (MIC)	Chloramphenicol 4.0 μg/mL (MIC)	[71]
		Well diffusion/*V. anguillarum*	32.0 μg/mL (MIC)	Chloramphenicol 1.0 μg/mL (MIC)	[71]
		Well diffusion/*V. harveyi*	0.5 μg/mL (MIC)	Chloramphenicol 8.0 μg/mL (MIC)	[71]
Chlorohydroasperlactone A (**100**)	Antibacterial	Well diffusion/*A. hydrophilia*	16.0 μg/mL (MIC)	Chloramphenicol 4.0 μg/mL (MIC)	[71]
		Well diffusion/*V. anguillarum*	16.0 μg/mL (MIC)	Chloramphenicol 1.0 μg/mL (MIC)	[71]
		Well diffusion/*V. harveyi*	16.0 μg/mL (MIC)	Chloramphenicol 8.0 μg/mL (MIC)	[71]
Asperochrin A (**104**)	Antibacterial	Well diffusion/*A. hydrophilia*	8.0 μg/mL (MIC)	Chloramphenicol 4.0 μg/mL (MIC)	[71]
		Well diffusion/*V. anguillarum*	16.0 μg/mL (MIC)	Chloramphenicol 1.0 μg/mL (MIC)	[71]
		Well diffusion/*V. harveyi*	8.0 μg/mL (MIC)	Chloramphenicol 8.0 μg/mL (MIC)	[71]
Aspyrone (**117**)	Cytotoxicity	CTG/MV-4-11	2.54 µM (IC_50_)	Adramycin 0.16 µM (IC_50_)	[31]
		CTG/K562	5.22 µM (IC_50_)	Adramycin 0.02 µM (IC_50_)	[31]
		CTG/A673	8.55 µM (IC_50_)	Adramycin 0.13 µM (IC_50_)	[31]
		CTG/N87	4.57 µM (IC_50_)	Adramycin 0.05 µM (IC_50_)	[31]
		CTG/H1299	5.83 µM (IC_50_)	Adramycin 0.49 µM (IC_50_)	[31]
		CTG/HUCCT1	9.79 µM (IC_50_)	Adramycin 0.05 µM (IC_50_)	[31]
		CTG/B16F10	5.89 µM (IC_50_)	Adramycin 0.02 µM (IC_50_)	[31]
		CTG/Karpass299	2.57 µM (IC_50_)	Adramycin 0.39 µM (IC_50_)	[31]
		CTG/HepB3	5.48 µM (IC_50_)	Adramycin 17.58 µM (IC_50_)	[31]
		CTG/HA431	5.92 µM (IC_50_)	Adramycin 0.17 µM (IC_50_)	[31]
		CTG/143B	6.32 µM (IC_50_)	Adramycin 0.10 µM (IC_50_)	[31]
		CTG/MKN-45	5.79 µM (IC_50_)	Adramycin 0.20 µM (IC_50_)	[31]
		CTG/H1975	2.99 µM (IC_50_)	Adramycin 0.09 µM (IC_50_)	[31]
		CTG/Hl-60	6.89 µM (IC_50_)	Adramycin 0.21 µM (IC_50_)	[31]
		CTG/DU145	5.61 µM (IC_50_)	Adramycin 0.05 µM (IC_50_)	[31]
		CTG/SPC-A1	9.51 µM (IC_50_)	Adramycin 0.19 µM (IC_50_)	[31]
		CTG/HEK-293F	50.35 µM (IC_50_)	Adramycin 0.05 µM (IC_50_)	[31]
		CTG/L02	14.20 µM (IC_50_)	Adramycin 0.10 µM (IC_50_)	[31]
Chlorohydroaspyrone A (**118**)	Antibacterial	Well diffusion/*A. hydrophilia*	16.0 μg/mL (MIC)	Chloramphenicol 4.0 μg/mL (MIC)	[71]
		Well diffusion/*V. anguillarum*	32.0 μg/mL (MIC)	Chloramphenicol 1.0 μg/mL (MIC)	[71]
		Well diffusion/*V. harveyi*	16.0 μg/mL (MIC)	Chloramphenicol 8.0 μg/mL (MIC)	[71]
Chlorohydroaspyrone B (**119**)	Antibacterial	Well diffusion/*A. hydrophilia*	16.0 μg/mL (MIC)	Chloramphenicol 4.0 μg/mL (MIC)	[71]
		Well diffusion/*V. anguillarum*	32.0 μg/mL (MIC)	Chloramphenicol 1.0 μg/mL (MIC)	[71]
		Well diffusion/*V. harveyi*	32.0 μg/mL (MIC)	Chloramphenicol 8.0 μg/mL (MIC)	[71]
Viomellein (**132**)	Cytotoxicity	MTT/L5178Y	5.3 µM (IC_50_)	Kahalalide F 4.3 µM (IC_50_)	[17]
		MTT/A2780	5.0 µM (IC_50_)	Cisplatin 2.2 µM (IC_50_)	[17]
(22*E*,24*R*)-ergosta-4,6,8(14),22-tetraen-3-one (**143**)	Anti-Parkinson’s disease	MPP^+^--induced SH-SY5Y cells	35.71 µM (EC_50_)	Levodopa 2.06 µM (EC_50_)	[24]
Ochrasterone (**150**)	Antioxidant	DPPH	189.10 µM (IC_50_)	BHT 91.35 µM (IC_50_)	[21]
		ABTS	60.21 µM (IC_50_)	Trolox 101.23 µM (IC_50_)	[21]
		FRAP	57.20 µM (IC_50_)	Trolox 1.80 µM (IC_50_)	[21]
Gymnasterone D (**151**)	Anti-Parkinson’s disease	MPP^+^--induced SH-SY5Y cells	38.75 µM (EC_50_)	Levodopa 2.06 µM (EC_50_)	[24]
Isocyathisterol (**152**)	Anti-Parkinson’s disease	MPP^+^--induced SH-SY5Y cells	46.18 µM (EC_50_)	Levodopa 2.06 µM (EC_50_)	[24]
Herbarulide (**153**)	Anti-Parkinson’s disease	MPP^+^--induced SH-SY5Y cells	37.67 µM (EC_50_)	Levodopa 2.06 µM (EC_50_)	[24]
Demethylincisterol A2 (**154**)	Anti-Parkinson’s disease	MPP^+^--induced SH-SY5Y cells	49.53 µM (EC_50_)	Levodopa 2.06 µM (EC_50_)	[24]
Clavatol (**156**)	Antioxidant	DPPH	30.0 µM (IC_50_)	l-ascorbic acid 20.0 μM (IC_50_)	[72]
Di-(2-ethylhexyl) phthalate (**159**)	Anti-Parkinson’s disease	MPP^+^--induced SH-SY5Y cells	80.21 µM (EC_50_)	Levodopa 2.06 µM (EC_50_)	[24]

## Data Availability

Not applicable.

## Abbreviations

786-O: Human renal carcinoma cell lines; 143B: human bone osteosarcoma cell line; A431: epidermoid carcinoma cell line; A549: human lung adenocarcinoma epithelial cell line; A673: rhabdomyoma cell line; A2780: human ovarian cancer cells line; A2780/DDP: human ovarian mutp53/bc12+ cancer cells line; A2780/Tax: human ovarian cancer taxol-resistant cells line; ABTS: 2,2′-Azinobis-(3-ethylbenzthiazoline-6-sulphonate); ACHN: human renal carcinoma cell lines; ADMET: absorption, distribution, metabolism, excretion, and toxicity; B16F10: highly metastatic mouse melanoma cell line; BGC-823: human gastric carcinoma cell line; BHT: butylated hydroxytoluene; BT474: hormone-sensitive breast cancer cell line; BV-2: microglia cells; CCK-8: cell counting kit-8; CD: circular dichroism; CH_2_Cl_2_: dichloromethane; CPE: cytopathic effect; CTG: cell Titer Glo; DPPH: 1,1-Diphenyl-2-picrylhydrazyl; DU145: human prostate carcinoma cell line; EC_50_: half maximal effective concentration; ECD: electronic circular dichroism; ELISA: enzyme-linked immunosorbent assay; EtOAc: ethyl acetate; FRAP: ferric reducing ability of plasma; GFPMA: green fluorescent protein microplate assay; GSH: glutathione; H1299: human non-small cell lung carcinoma cell line; HCC1954: grade 3 invasive ductal carcinoma cell line; H1975: human non-small cell lung carcinoma with L858R and T790M mutation cell line; H_2_O: water; H_2_O_2_: hydrogen peroxide; HCT-116: human colon cancer cell line; HCT-116/mdr+: human colon overexpress mdr+ cancer cell line; HCT-116/topo: human colon resistant to etoposide cancer cell line; HCV: hepatitis C virus; HEK-293F: human embryonic kidney-293F cell line; HepG2: human hepatocellular liver carcinoma cell line; Hep3B: human liver cancer cell line; HeLa: human cervical epithelioid carcinoma cell line; HL-60: human promyelocytic leukemia cell line; HPLC: high-performance liquid chromatography; HUCCT1: bile duct carcinoma cell line; Huh-7: human male hepatoma cell line; IC_50_: half-maximal inhibitory concentration; IC_90_: the concentration that will inhibit 90% of the virions; ICD: induced circular dichroism; IL-1β: interleukin-1beta; K562: human erythroleukemic cell line; Karpass299: human T cell lymphoma cell line; L02: human liver cell line; IR: infrared; LPS: lipopolysaccharide; LNCaP: human prostatic-testosterone-sensitive cancer cell line; L5178Y: mouse lymphoma cell line; LX-1: human lung sensitive cancer cell line; MCF-7: human breast adenocarcinoma cell line; MDA-MB-231: human breast cancer cell line; MDA-MB-468: basal breast cancer cell line; MeOH: methanol; MIC: minimum inhibitory concentrations; MKN-45: human gastric cancer cell line; Molt-4: human T lymphoblast cell line; MS: mass spectrometry; MPP^+^: 1-Methyl-4-phenylpyridinium; MTT: 3-(4,5-Dimethylthiazol-2-yl)-2,5-diphenyltetrazolium bromide; MV-4-11: biphenotypic B myelomonocytic leukemia cell line; N87: gastric carcinoma cell line; NADH: nicotinamide adenine dinucleotide; NCI-H460: human non-small cell lung cancer cell line; NF-κB: nuclear factor kappa-light-chain-enhancer of activated B cells; NMR: nuclear magnetic resonance; NO: nitric oxide; OSRC-2: human renal carcinoma cell lines; PC-3: human prostatic-testosterone-independent cell line; ROS: reactive oxygen species; RP-18: reversed phase-18; SH-SY5Y: thrice-cloned subline of the neuroblastoma cell line; SRB: sulforhodamine B; SiO_2_ CC: silica gel column chromatography; SKBR-3: human breast estradiol-independent cancer cell line; SMMC-7721: human hepatoma cell line; SMP: submitochondrial particles; SPA: scintillation proximity assay; SPC-A1: human lung cancer cell line overexpressing maspin cell line; SW1990: human pancreatic cancer cell line; TLC: thin layer chromatography; TNF-α: rumor necrosis factor alpha; Trolox: 6-Hydroxy-2,5,7,8-tetramethylchroman-2-carboxylic acid; U87: glioblastoma cell line; U251: glioblastoma cell line; U937: pro-monocytic, human myeloid leukaemia cell line; XRD: X-ray diffraction.
